# The phytohormone abscisic acid enhances remyelination in mouse models of multiple sclerosis

**DOI:** 10.3389/fimmu.2024.1500697

**Published:** 2024-12-17

**Authors:** Femke Van Gaever, Fleur Mingneau, Sam Vanherle, Yasmine Driege, Mira Haegman, Elien Van Wonterghem, Junhua Xie, Roosmarijn E. Vandenbroucke, Jerome J. A. Hendriks, Rudi Beyaert, Jens Staal

**Affiliations:** ^1^ VIB-UGent Center for Inflammation Research, VIB, Ghent, Belgium; ^2^ Department of Biomedical Molecular Biology, Ghent University, Ghent, Belgium; ^3^ Department of Immunology and Infection, Biomedical Research Institute, Hasselt University, Diepenbeek, Belgium; ^4^ University MS Center Hasselt, Pelt, Belgium; ^5^ Department of Biochemistry and Microbiology, Ghent University, Ghent, Belgium

**Keywords:** abscisic acid, remyelination, macrophages, microglia, multiple sclerosis, myelin, neuroinflammation

## Abstract

**Introduction:**

Over the past few decades, there has been a sudden rise in the incidence of Multiple Sclerosis (MS) in Western countries. However, current treatments often show limited efficacy in certain patients and are associated with adverse effects, which highlights the need for safer and more effective therapeutic approaches. Environmental factors, particularly dietary habits, have been observed to play a substantial role in the development of MS. In this study, we are the first to investigate the potential protective effect of the phytohormone abscisic acid (ABA) in MS. ABA, which is abundant in fruits such as figs, apricots and bilberries, is known to cross the blood-brain barrier and has demonstrated neuroprotective effects in conditions like depression and Alzheimer's disease.

**Methods:**

In this study, we investigated whether ABA supplementation enhances remyelination in both *ex vivo* and *in vivo* mouse models.

**Results:**

Our results indicated that ABA enhanced remyelination and that this enhanced remyelination is associated with increased lipid droplet load, reduced levels of degraded myelin, and a higher abundance of F4/80+ cells in the demyelinated brain of mice treated with ABA. In *in vitro* models, we further demonstrated that ABA treatment elevates lipid droplet formation by enhancing the phagocytic capacity of macrophages. Additionally, in a mouse model of microglial activation, we showed that ABA-treated mice maintain a less inflammatory microglial phenotype.

**Conclusion:**

Our findings highlight a crucial role for macrophages and microglia in enabling ABA to enhance the remyelination process. Furthermore, ABA’s ability to improve remyelination together with its ability to reduce microglial activation, make ABA a promising candidate for modulating macrophage phenotype and reducing neuroinflammation in MS.

## 1 Introduction

Multiple sclerosis (MS) is a chronic inflammatory disorder affecting the central nervous system (CNS), with an estimated global number of 2.8 million people living with the disease (1). MS is characterized by repeated episodes of inflammatory demyelination, a process where the protective myelin sheaths surrounding axons and neurons are lost, leaving axons and neurons more susceptible to degeneration (2–4). Failure of remyelination, the restoration of damaged myelin sheaths, underlies the progressive nature of MS, resulting in chronically demyelinated plaques. The dominant hypothesis states that remyelination is hindered due to inadequate recruitment of oligodendrocyte precursor cells (OPCs) to the lesion site, coupled with a reduced ability of these cells to differentiate into mature, myelinating oligodendrocytes (2, 5). Emerging evidence indicates that impaired differentiation of OPCs is influenced by a dysfunctional innate immune response in the CNS (6–13). Demyelinating lesions contain abundant peripheral macrophages and CNS-derived microglia (14–20), which perform dual functions, executing both beneficial, disease-resolving, and detrimental, disease-promoting, effects. Phagocytes facilitate remyelination by clearing damaged myelin and increasing production of neurotrophic factors (21–25), but they also contribute to neuroinflammation, demyelination and neurodegeneration (12, 26). Substantial evidence indicates that the inflammatory and reparative functions of phagocytes are determined by their intracellular lipid load (12). Initially, myelin uptake skews phagocytes toward an immunosuppressive, reparative phenotype, accompanied by the production of neurotrophic factors (8, 12, 14, 21–23, 26–28). However, sustained internalization of myelin leads to the formation of inflammatory foamy phagocytes that hinder CNS repair (11–14, 20). Directing macrophages towards a disease-resolving phenotype and restoring their capacity to degrade and eliminate myelin-derived cholesterol is regarded as a promising strategy for limiting the progression of MS and promoting remyelination (12, 29–36). Since internalized myelin skews macrophages towards an anti-inflammatory phenotype by activating the liver X receptor (LXR) and peroxisome proliferator-activated receptor γ (PPARγ) (22, 37, 38), modulation of these signaling pathways could also be a promising strategy to enhance repair (9, 30). Overall, treatments that impact both immune responses and repair mechanisms are expected to be the most effective in promoting remyelination.

Dietary components are (re)surfacing as promising candidates for therapeutic use in MS due to their tendency to cause fewer adverse effects and potentially even outperform synthetic compounds in terms of biological and pharmacological activities (reviewed in (39)). Many dietary components are known for their influence on neuroinflammation and macrophage phenotype alteration (reviewed in (40)). Flavonoids, the largest phytonutrient family, are increasingly being recognized for their ability to exhibit anti-inflammatory effects on macrophages (41–45). However, other secondary metabolites, such as phytohormones might also provide therapeutic benefits (46). In this study, we highlight abscisic acid (ABA), a phytohormone found in significant concentrations in fruits such as figs, bilberries and apricots, as a promising candidate for MS treatment. ABA is known for its involvement in diverse immune and inflammatory reactions (46) and dietary administration of ABA to both animals and humans can offer protection against conditions such as colitis (47) and type 2 diabetes (48–51). Furthermore, ABA is able to cross the blood-brain-barrier and shows protective effects in animal models of depression (52–54), high-fat diet (HFD)-induced neuroinflammation (55, 56) and Alzheimer’s disease (57). In these conditions, ABA was shown to suppress expression of pro-inflammatory genes, to increase expression of anti-inflammatory cytokines (47, 56–58) and to rescue the increase in microglia activation (57, 59, 60). Mechanistically, it was demonstrated that ABA regulates inflammation via the ligand binding domain-independent activation of PPARγ (61), acting through a lanthionine synthetase component C-like protein 2 (LANCL2) - PPARγ axis (51, 58, 59, 61–63). Given the anti-inflammatory effects of ABA in neuroinflammation and Alzheimer’s disease in combination with its capacity to activate PPARγ, we hypothesized that ABA could improve remyelination through modulation of macrophage phenotype and neuroinflammation in experimental models of MS.

## 2 Materials and methods

### 2.1 Antibodies and chemical reagents

Abscisic acid (GoldBio) was dissolved in 100 mM NaOH to create a 75 mM stock solution and stored at -20°C. Further dilutions were made in RPMI1640 medium (Gibco). In the mouse model of microglial activation, lipopolysaccharide (LPS; L5886, Sigma-Aldrich) was administered at a dose of 3 mg/kg body weight. The following antibodies were used for immunofluorescence: rabbit anti-myelin basic protein (MBP; 1:250; MAB386, Sigma-Aldrich; brain cryosections), rat anti-MBP (1:250; MCA409S, Sigma-Aldrich; cerebellar brain slices), rabbit anti-myelin basic protein (dMBP; 1:2000; AB5864, Merck; cerebellar brain slices), rabbit anti-neurofilament (NF; 1:1000; ab8135, Abcam), rat anti-F4/80 (1:100; MCA497G, Bio-Rad), rabbit anti-iNOS (1:100; ab15323, Abcam), mouse anti-CC1 (1:100; ab16794, Abcam), and goat anti-Olig2 (1:100; AF2418, R&D Systems). Appropriate secondary antibodies were obtained from Invitrogen.

### 2.2 Mice

For mouse bone marrow derived macrophage (BMDM) experiments, C57BL/6J mice were bred at the Biomedical Research Institute of Hasselt University. For *in vivo* experiments, male and female wild-type C57BL/6J mice (8-12 weeks old) were obtained from Janvier (France) and maintained under specific pathogen-free conditions. Mice were acclimatized for 2 weeks before initiation of the experiments. The mice were maintained on a 12-hour light/dark cycle with free access to water. Mice were fed either a standard chow diet or a specially formulated diet (abscisic acid-supplemented diet; 400 mg/kg, Research diets Inc.) as described previously (49). All animal procedures were conducted in accordance with the institutional guidelines and approved by the Ethical Committee for Animal Experiments of the VIB site at Ghent University Faculty of Sciences (EC2022-087, EC2023-029, EC2024-005) or the Ethical Committee for Animal Experiments of Hasselt University (202408K; *ex vivo* use). The number of animals was determined by power analysis using G*power software.

### 2.3 *In vivo* cuprizone-induced demyelination

Acute demyelination was induced as described previously (36). Briefly, 9-11-week-old male mice were fed ad libitum a diet containing 0.3% cuprizone (bis[cyclohexanone]oxaldihydrazone; Sigma-Aldrich) mixed into powdered chow, either with the standard formulation or supplemented with abscisic acid (400 mg/kg), for 5 weeks. Following withdrawal of the cuprizone diet, mice were fed powdered standard chow or ABA-supplemented chow for 1 week. Male mice were used because they show a more consistent demyelination response than female mice. Mice were euthanized at 5 weeks (5 wk) or after 1 week of recovery (5 + 1 wk) with intraperitoneal injection of ketamine (100 mg/mL) and xylazine (20 mg/mL) followed by transcardial perfusion. Blood and brain tissues were collected for ABA measurement, histological and biochemical analysis. In this experiment, groups were composed of 7-8 animals per condition.

### 2.4 Experimental autoimmune encephalomyelitis

Starting 1 week before immunization and throughout the experiment, female mice were fed ad libitum with either an ABA-supplemented diet (400 mg/kg) or a control diet. At the age of 11 weeks, experimental autoimmune encephalomyelitis (EAE) was induced. In this experiment, female mice were used because they show more reliable and uniform EAE development. Female C57BL/6J mice were sedated with isoflurane (4%) to minimize suffering and were immunized subcutaneously, according to manufacturer’s instructions (EK-2110; Hooke Laboratories), with 2 times 100 µL of recombinant human myelin oligodendrocyte glycoprotein peptide (MOG35–55) (~300ng) emulsified in complete Freund’s adjuvant containing Mycobacterium tuberculosis H37Ra. Directly after immunization and after 24 hours, mice were injected intraperitoneally with 100 µL of pertussis toxin (121 ng). A control group (no EAE) was included where mice were not immunized and received an injection with phosphate buffered saline (PBS) instead of pertussis toxin. This group allowed for better comparison of body weight and clinical scoring. Throughout the experiment, from the day of immunization until the day of sacrifice, mice were weighed daily and clinically scored for neurological signs of the disease according to the manufacturer’s mouse EAE scoring guide: 0: no clinical symptoms; 0.5: distal tail paralysis; 1: tail paralysis; 2: tail paralysis and partial hindlimb paralysis; 2.5: tail paralysis and dragging of hind legs; 3: complete hindlimb paralysis; 4: paralysis to the diaphragm; 5: death by EAE. Animal welfare and suffering of the mice was monitored daily. Mice were humanely euthanized when they had lost 20% of their initial body weight or at day 24. Euthanasia was performed using intraperitoneal injection of ketamine (100 mg/mL) and xylazine (20 mg/mL) followed by transcardial perfusion. In this experiment, groups were composed of 8 animals per condition.

### 2.5 Induction of low-grade LPS inflammation

Starting 1 week before injection and throughout the experiment, mice were fed ad libitum with either an ABA-supplemented diet (400 mg/kg) or a control diet. Mice were injected intraperitoneally with LPS (3.0 mg/kg body weight, i.p.) or PBS, as described previously (64). Body weight and temperature were checked 5 hours, 10 hours and 24 hours post-injection and mice were sacrificed at the 24 hours timepoint. Euthanasia was performed using intraperitoneal injection of ketamine (100 mg/mL) and xylazine (20 mg/mL) followed by transcardial perfusion. Blood and brain tissues were collected for ABA measurement, histological and biochemical analysis. In this experiment, groups were composed of 5 animals per condition.

### 2.6 Macrophage differentiation and treatment

BMDMs were isolated and differentiated as previously described (36). Briefly, tibial and femoral bone marrow suspensions from 9-12-week-old female wild-type C57BL/6J mice were cultured in 10 cm petri plates at a concentration of 10 x 10^6^ cells/plate. The cells were differentiated in bone marrow medium (RPMI1640 supplemented with 10% fetal calf serum (FCS, Gibco), 50 U/mL penicillin (Invitrogen), and 50 U/mL streptomycin (Invitrogen)) supplemented with 15% L929-conditioned medium (LCM). After differentiation, BMDMs were detached at 37°C with 10 mM EDTA in PBS (Gibco) and plated for experiments at 0.5 x 10^6^ cells/mL in bone marrow medium supplemented with 5% LCM (37°C, 5% CO_2_). Cells were treated daily with mouse myelin (100 μg/mL) for 24 hours (mye^24h^) or 72 hours (mye^72h^), and ABA (10 µM).

### 2.7 Cerebellar brain slice cultures

Cerebellar brain slices were obtained from C57BL/6 mouse pups at postnatal day 9 or 10 (P9 or P10), as described previously (65, 66). The brain slices were cultured in MEM medium (Thermo Fisher Scientific) supplemented with 25% horse serum (Thermo Fisher Scientific), 25% Hank’s balanced salt solution (Sigma‐Aldrich), 50 U/mL penicillin, 50 U/mL streptomycin, 1% Glutamax (Thermo Fisher Scientific), 12.5 mM HEPES (Thermo Fisher Scientific), and 1.45 g/L glucose (Sigma‐Aldrich). To induce demyelination, brain slices were treated with lysolecithin (LPC; 0.5 mg/mL, Sigma-Aldrich) for 16 hours at 3 days post isolation. After demyelination, brain slices were allowed to recover in culture medium for 1 day, followed by daily treatment with vehicle (PBS), ABA (1-10 μM) or PPARγ inhibitor (GW9662; 10µM; Sigma-Aldrich) for 1 week. For microglia depletion experiments, slices were treated with clodronate liposomes or empty liposomes (0.5 mg/mL; LIPOSOMA) for 24 hours, immediately after isolation.

### 2.8 Immunofluorescence microscopy and image analysis

Cerebellar brain slices were fixed in 4% paraformaldehyde (PFA) for 15 minutes at room temperature, while frozen brain cryosections were air dried and fixed in ice-cold acetone for 10 minutes at -20°C. For low permeabilization staining with MBP, cryosections were fixed in 4% PFA for 4 hours at 4°C and dehydrated using a sucrose gradient. Cerebellar brain slices were blocked with a buffer containing 5% normal horse serum and 0.3% Triton X-100 in PBS for 1 hour, while brain cryosections were treated with Dako protein block (Agilent) for 30 minutes and cryosections in the low permeabilization experiment were blocked with a buffer containing 1% normal goat serum, 0.5% BSA and 0.1% Tween. After blocking, brain slices and cryosections were incubated overnight at 4°C with primary antibodies. Following washing steps, they were exposed to the suitable secondary antibodies for 2 hours and 1 hour, respectively, at room temperature. Images of cerebellar brain slices were made on an LSM880 confocal microscope (Zeiss). For every brain slice at least 3 pictures were taken in different cerebellar lobes. The level of remyelination was quantified using the myelination index, computed as the ratio of the colocalized area of MBP and NF by the total NF area. Colocalization was calculated using the colocalize threshold plugin in ImageJ. dMBP staining was analyzed using ImageJ thresholder and denoted as percentage of total area. Images of brain cryosections were taken using a Leica DMi 8 microscope and LAS X Office software (Leica) and analyzed using the thresholder in ImageJ software. Measurements are shown as percentage of corpus callosum area. The researchers were blinded during sample processing.

### 2.9 Oil red O staining

Mouse BMDMs, cultured on glass cover slides, were fixed in 4% PFA for 15 minutes at room temperature. To visualize intracellular myelin degradation products, fixed BMDMs, unfixed frozen brain cryosections, and cerebellar brain slices were stained with 0.3% Oil Red O (ORO; Sigma-Aldrich) for 10 minutes. Cell nuclei were counterstained with hematoxylin. Images were captured using an Axio Scan.Z1 microscope (Zeiss) or an LSM880 confocal microscope (Zeiss) and analyzed with the QuPath software or the ImageJ software. For BMDMs, ORO stain was determined as percentage of cell area. For brain cryosections, ORO stain was determined as percentage of corpus callosum. For cerebellar brain slices, ORO stain was determined as percentage of total brain slice. The researchers were blinded during sample processing.

### 2.10 Transmission electron microscopy

For transmission electron microscopy (TEM) analysis, 1 mm-thick coronal brain sections were collected at approximately -2.7 mm from the bregma. From these sections, the corpus callosum was dissected, and the portion from the right hemisphere was used for TEM. These dissected samples were immersed in a fixative solution containing 2.5% glutaraldehyde and 4% PFA in 0.1 M sodium cacodylate buffer (pH 7.2). The samples were placed in a vacuum oven for 1 hour, then rotated for 3 hours at room temperature. The fixative solution was subsequently replaced with fresh fixative, and samples were left rotating overnight at 4°C. After washing, the samples were post-fixed overnight in 1% OsO_4_ with 1.5% K_3_Fe(CN)_6_ in 0.1 M sodium cacodylate buffer at 4°C. The samples were rinsed in distilled water and left in 1% uranyl acetate for 1 hour for bulk staining. Samples were then dehydrated through a graded ethanol series, followed by embedding in Spurr’s resin. Ultrathin sections were cut perpendicular to the corpus callosum using an ultramicrotome (Leica EM UC6) and were transferred to 0.7% formvar-coated copper grids (Aurion). Grids were viewed with a JEM-1400plus transmission electron microscope (JEOL, Tokyo, Japan) operating at 80 kV. ImageJ was used to calculate the g-ratio (the ratio of the inner axonal diameter to the total outer diameter) using 8-10 images per animal. MyelTracer software (67) was used to count the number of myelinated axons. The researchers were blinded during sample processing.

### 2.11 Microglia 3D reconstruction

For 3D reconstruction of microglia, dissected brains were fixed overnight in 4% PFA and embedded in 5% agarose. 50 μm thick sagittal sections were cut using a vibratome (Leica). Brain sections were blocked with a buffer containing 5% normal goat serum and 0.5% Triton X-100 in PBS for 1 hour. After blocking, brain sections were stained overnight with anti-IBA1 antibody at 4°C, followed by secondary antibodies for 2 hours at room temperature. Z-stack images were taken in the CA1 region of the hippocampus with an LSM 780 microscope (Zeiss). The 3D reconstructions and measurements were done by the filament tracing algorithm from Arivis Vision 4D software (Zeiss). The researchers were blinded during sample processing.

### 2.12 Quantitative reverse transcription PCR

For BMDM experiments, cells were treated with ABA (10 μM) and stimulated with myelin (100 µg/mL) for 24 or 72 hours. Cell lysis was performed using Qiazol (Qiagen). For mouse experiments, corpus callosum tissue was lysed using Trizol (Invitrogen). RNA from cells and tissue was extracted using the RNeasy Mini Kit (Qiagen) and Total RNA Mini Kit (Aurum), respectively. RNA concentration and quality were determined with a Nanodrop spectrophotometer (Isogen Life Science). DNase I treatment was performed on RNA using the DNAse I recombinant kit (4716728001, Sigma-Aldrich). cDNA synthesis was conducted using the SensiFast cDNA Synthesis Kit (GC Biotech) according to the manufacturer’s instructions. qPCR was performed on a Real-Time PCR system (Lightcycler 480, Roche) using the SensiFast SYBR No-Rox Kit containing 1x SYBR green (GC Biotech), 0.3 μM primers (Integrated DNA Technologies), 2-8 ng cDNA and nuclease-free water. mRNA expression was analyzed using qbase+ software version 3.2 (Biogazelle). GeNorm was used to select stable housekeeping genes. A list of primer sequences is provided in **Supplementary Table S1**.

### 2.13 Chemokine and cytokine measurement

Chemokines and cytokines were measured in plasma using a Bio-Plex assay for tumor necrosis factor α (TNFα) (12002444; Bio-Rad), interferon γ (IFNγ) (12002438; Bio-Rad), interleukin 6 (IL6) (12002241; Bio-Rad), interleukin 10 (IL10) (12002242; Bio-Rad), C-C motif chemokine 5 (CCL5) (12002256; Bio-Rad) and monocyte chemoattractant protein 1 (MCP1) (12002441; Bio-Rad) according to the manufacturer’s instructions. Signals were measured on a Bio-Plex 200 system (Bio-Rad).

### 2.14 Myelin isolation, phagocytosis and lipid droplet staining

Myelin was purified from postmortem mouse brain tissue by means of density gradient centrifugation, as described previously (68). Myelin protein concentration was determined by using the BCA protein assay kit (Thermo Fisher Scientific), according to manufacturer’s guidelines. Cells were treated with 100 μg/mL myelin. To evaluate the ability and extent of myelin phagocytosis, cells were pre-treated with vehicle (PBS) or ABA (10 µM) for 24 hours and were subsequently exposed to 100 µg/mL myelin that was fluorescently labeled with pHrodo™ intracellular pH indicator dye (P35372; Invitrogen) in presence or absence of ABA for 1.5 hours. Fluorescence intensity was analyzed using an LSRFortessa (BD Biosciences). BMDMs were stained for intracellular lipid droplet load by 15-minute incubation with BODIPY (493/503) (D3922, Thermo Fisher Scientific) at 37°C. The LSRFortessa (BD Biosciences) was used to quantify cellular fluorescence.

### 2.15 Cholesterol measurements

Cholesterol levels of BMDMs were defined by using the Amplex Red Cholesterol Assay kit (A12216; Thermo Fisher) according to the manufacturer’s instructions. BMDMs were treated with myelin for 24 hours or 72 hours in presence or absence of ABA (10µM). Fluorescence was measured using the Infinite 200 pro microplate reader (Tecan).

### 2.16 ABA extraction from brain

ABA extraction for measurement in brain samples was performed as described before by Maixner et al. (69). Briefly, snap frozen brain tissue was homogenized with ice-cold 80% methanol and shaken for 24 hours at 4°C. The supernatant was collected, additional methanol was added to the pellet, and samples were shaken for 1 hour at 4°C. The total supernatant was collected and evaporated on a rotary evaporator. Petroleum ether was added to the liquid and mixed. After the liquid became layered, the top layer of petroleum ether was removed by pipetting and the bottom methyl alcohol layer was collected and evaporated again. After methanol had completely evaporated, the pellet was resuspended in 50 µL of 10% methanol.

### 2.17 ABA measurements

ABA measurements were performed using a highly sensitive biosensor, engineered by our lab (70). Briefly, this biosensor is a HEK 293T cell line stably transfected with a plasmid containing the *Arabidopsis* PYL1^H87P^ mutant ABA receptor coupled to a VP16 activation domain, and the ABA coreceptor ABI1 coupled to a GAL4 binding domain and a plasmid containing a luciferase reporter gene. Presence of ABA brings the VP16 activation domain to a GAL4-dependent promotor, leading to luciferase gene expression. To measure ABA in serum or brain samples, reporter cells were seeded at 10.000 cells per well in 96-well plates. One day post seeding, samples were added at a dilution of 1:10 when the medium was refreshed. Positive controls of 80, 40, 20, 10, 5, 2.5 and 1.25 nM ABA, as well as a negative control were used to create a standard concentration curve. This standard curve was spiked with ABA-negative serum or brain lysate to correct for sample type induced effects. After 24 hours, cells were lysed in luciferase lysis buffer (25mM Tris-phosphate pH7.8, 2 mM DTT, 2mM CDTA, 10% glycerol, 1% Triton X-100). To cell lysates, D-luciferin (E1605, Promega) was added. Luminescence signals were measured in triplicate using the Glomax^®^ 96 Microplate Luminometer (Promega).

### 2.18 Statistical analysis

Statistical analyses and data visualization were performed using Prism (Graphpad, La Jolla, CA). Data are presented as mean ± standard error of the mean (SEM). The number of biological replicates is indicated by dots in the figure and denoted as “n” in the legend. Data distribution and variance characteristics were considered for statistical testing. For normally distributed datasets, an ordinary one-way ANOVA with correction for multiple testing or a two-tailed unpaired Student’s t-test was used. Statistical significance was defined as p<0.05, with significance levels indicated as *p<0.05, **p<0.01, ***p<0.001, and ****p<0.00001. No randomization was done, and the investigator was not blinded to the mouse group allocation. The sample size was determined by power analysis using G*power software. Outlier data points were removed on the basis of the robust regression and outlier removal (ROUT) method.

## 3 Results

### 3.1 Abscisic acid improves remyelination in *ex vivo* and *in vivo* mouse models of demyelination

**Figure 1:**
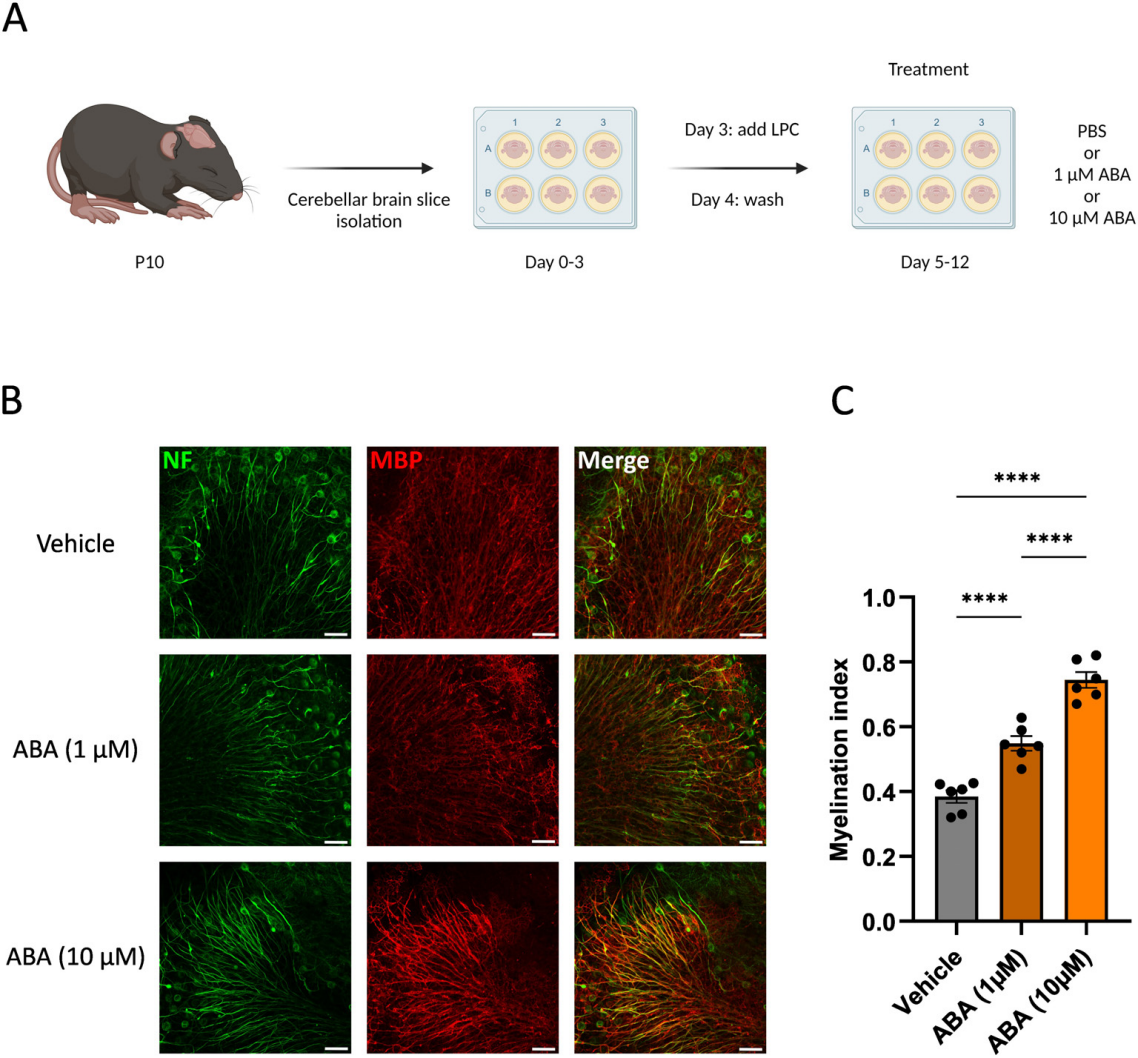
ABA improves remyelination in the cerebellar brain slice model. **(A)** Schematic representation showing the isolation and culture of cerebellar brain slices as well as their stimulation with vehicle (PBS), or ABA. LPC = lysolecithin, demyelinating compound. Created with biorender.com. **(B)** Representative images of orthogonal projections of immunofluorescent MBP/NF stains of cerebellar brain slices treated with vehicle or ABA. Scale bars, 50 μm. **(C)** Relative number of MBP+ NF+ axons out of total NF+ axons in cerebellar brain slices treated with vehicle or ABA (n = 6). Results are pooled from two independent experiments. Each dot represents one slice. Data are represented as mean ± SEM and statistically analyzed using a one-way ANOVA with correction for multiple testing. ****p<0.0001.

**Figure 2:**
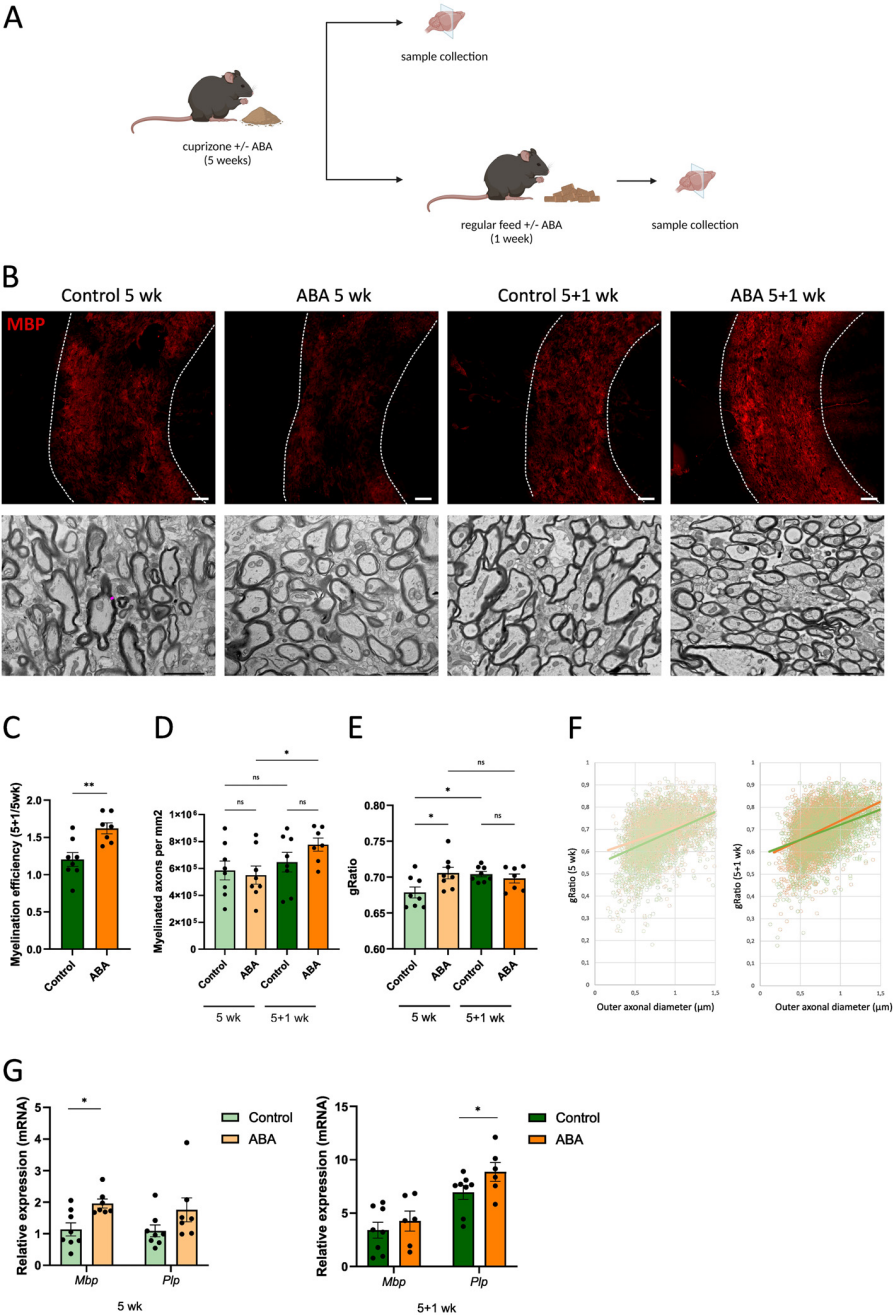
ABA improves remyelination in the cuprizone model. **(A)** Schematic representation showing the experimental set-up used to assess the impact of ABA on remyelination in the cuprizone model. Created with biorender.com. **(B)** Representative images of immunofluorescence myelin (MBP) staining and transmission electron microscopy analysis of the corpus callosum from mice fed control diet or ABA (400 mg/kg)-supplemented diet, after cuprizone-induced demyelination (5 wk) and during remyelination (5 + 1wk). In the upper panel, the outer border of the corpus callosum is demarcated by the dotted line. In the lower panel, the pink line represents an example of myelin sheath thickness, measured by the difference between the inner and outer axonal diameters. Scale bars, 200 μm (MBP) and 2 μm (TEM). **(C)** Quantification of the remyelination efficacy (calculated by dividing the percent myelination at 5 + 1 weeks by the percent myelination at 5 weeks, using the MBP staining) in corpus callosum from control-fed and ABA-fed mice (n = 7-8 animals; 2 images per animal). **(D-F)** Mean number of myelinated axons per mm^2^ **(D)**, g-ratio (the ratio of the inner axonal diameter to the total outer diameter) **(E)**, and g-ratio as a function of outer axonal diameter **(F)** were analyzed in corpus callosum from control-fed and ABA-fed mice after 5 weeks and 5 + 1 weeks (n = 7–8 animals; for each animal 8 pictures were analyzed amounting to a total of 169–489 axons). **(G)** mRNA expression of *Mbp* and *Plp* in the corpus callosum from control-fed and ABA-fed mice after 5 weeks and 5 + 1 weeks (n = 7–8 animals). Each dot represents one mouse. Data are represented as mean ± SEM and statistically analyzed using a one-way ANOVA with correction for multiple testing. *p<0.05, **p<0.01.

**Figure 3:**
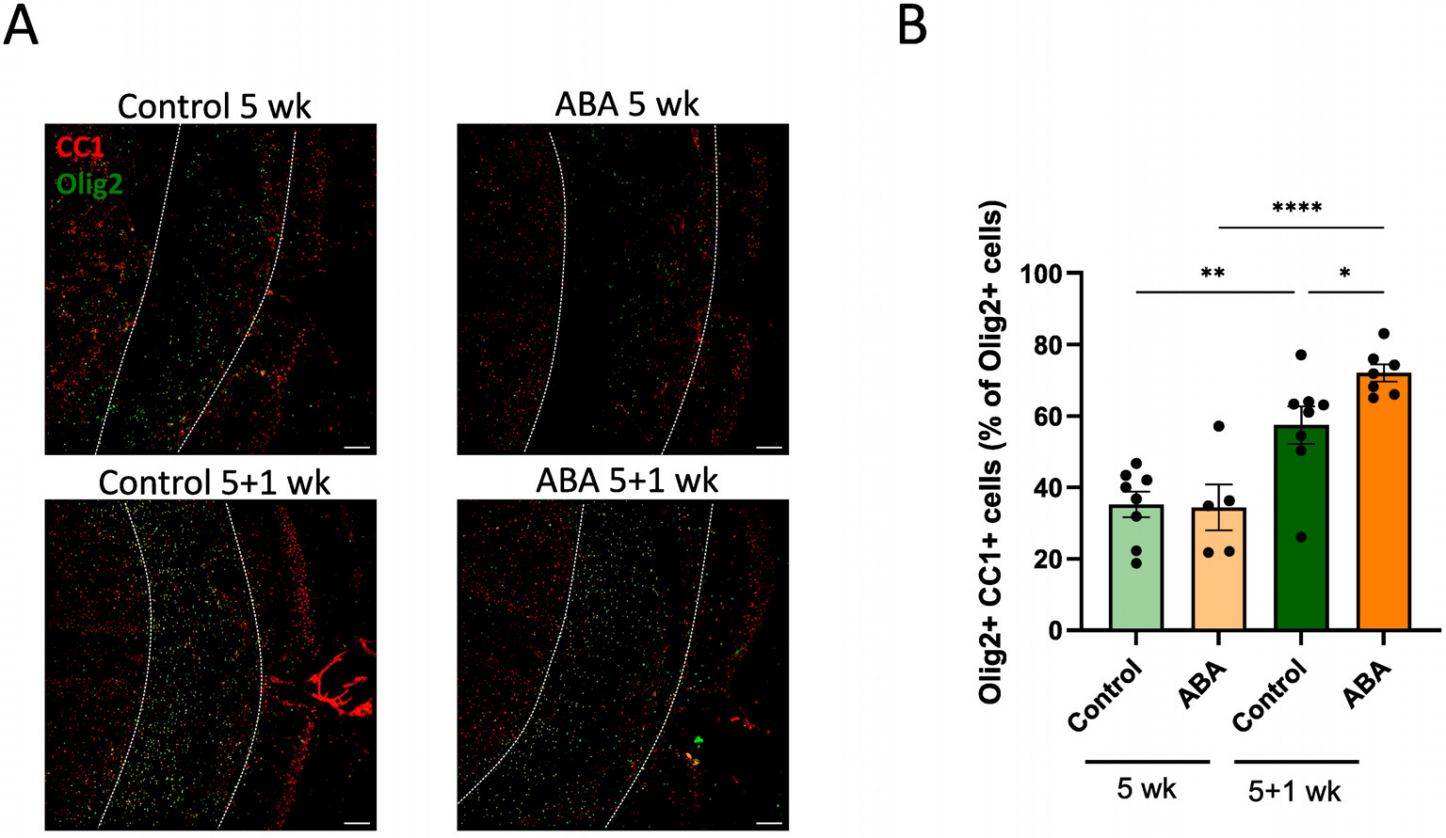
ABA increases the abundance of mature oligodendrocytes. **(A)** Representative images of immunofluorescent Olig2/CC1 staining of the corpus callosum from mice fed with control diet or ABA-supplemented diet after cuprizone-induced demyelination (5 wk) and during remyelination (5 + 1 wk). The outer border of the corpus callosum is demarcated by the dotted line. Scale bars, 200 μm. **(B)** Quantification of the percentage Olig2+ CC1+ cells out of total Olig2+ cells in the corpus callosum from control-fed and ABA-fed mice (5 + 1 wk) (n = 7-8 animals; 2 images per animal). Each dot represents one mouse. Data are represented as mean ± SEM and statistically analyzed using a one-way ANOVA with correction for multiple testing. *p<0.05, **p<0.01, ****p<0.0001.

To determine the impact of ABA on remyelination, *ex vivo* and *in vivo* mouse models were used. In the *ex vivo* model, cerebellar brain slices were demyelinated with lysolecithin and subsequently exposed to ABA (experimental design in **Figure 1A**). Fluorescent staining showed increased colocalization of myelin (MBP) and axons (NF) in brain slices treated with ABA, which was quantified by the myelination index (p<0.0001). This increased colocalization suggests more efficient axonal remyelination in ABA-exposed brain slices (**Figures 1B, C**). To evaluate the *in vivo* significance of ABA’s reparative capacity, a cuprizone-induced de- and remyelination model was employed in mice. The mice were given either a control diet or an ABA-supplemented diet, resulting in increased levels of ABA detectable in their serum (p<0.0002 and p<0.003 respectively) (**Supplementary Figure S1**). Cuprizone feeding leads to reproducible, prominent demyelination in various CNS regions, particularly in the corpus callosum (71). Mice were fed a control or ABA diet supplemented with 0.3% cuprizone for 5 weeks, followed by 1 week on a diet without cuprizone, during which spontaneous remyelination occurred. The mice were pathologically characterized after demyelination (5 weeks) and during remyelination (5 + 1 weeks) (experimental design in **Figure 2A**). One week after cuprizone withdrawal, ABA-fed mice displayed increased myelination efficiency in the CC, indicated by the ratio of MBP at 5 + 1 weeks compared to 5 weeks (p<0.004) (**Figures 2B, C**). Accordingly, TEM demonstrated that during the remyelination phase, ABA-fed mice had a significantly higher number of myelinated axons in the corpus callosum compared to the demyelination phase (p<0.03) (**Figures 2B–D**). However, no overall differences were observed in the g-ratio (ratio of the inner axonal diameter to the total outer diameter) during remyelination (**Figures 2B, E**). Interestingly, when axon size was considered, a higher g-ratio was observed in large-diameter axons of ABA-fed mice during remyelination (**Figure 2F**). During demyelination, a similar effect was noted in small-diameter axons (**Figures 2E, F**). These measurements suggest the presence of more myelinated axons but with a thinner myelin sheath. In support of these findings, ABA-fed mice exhibited a significantly increased mRNA expression of *Mbp* after demyelination (5 weeks) (p<0.02) and proteolipid protein (*Plp)* expression was significantly elevated during remyelination (p<0.03) (**Figure 2G**). Consistent with these findings, the corpus callosum of ABA-fed mice showed an increased abundance of Olig2+ CC1+ mature oligodendrocytes during remyelination compared to control-fed mice (p<0.04) (**Figure 3**). These results indicate that ABA promotes remyelination *in vivo* by enhancing differentiation of oligodendrocyte precursor cells.

### 3.2 Abscisic acid increases lipid droplet formation and promotes remyelination in a phagocyte-dependent manner

**Figure 4:**
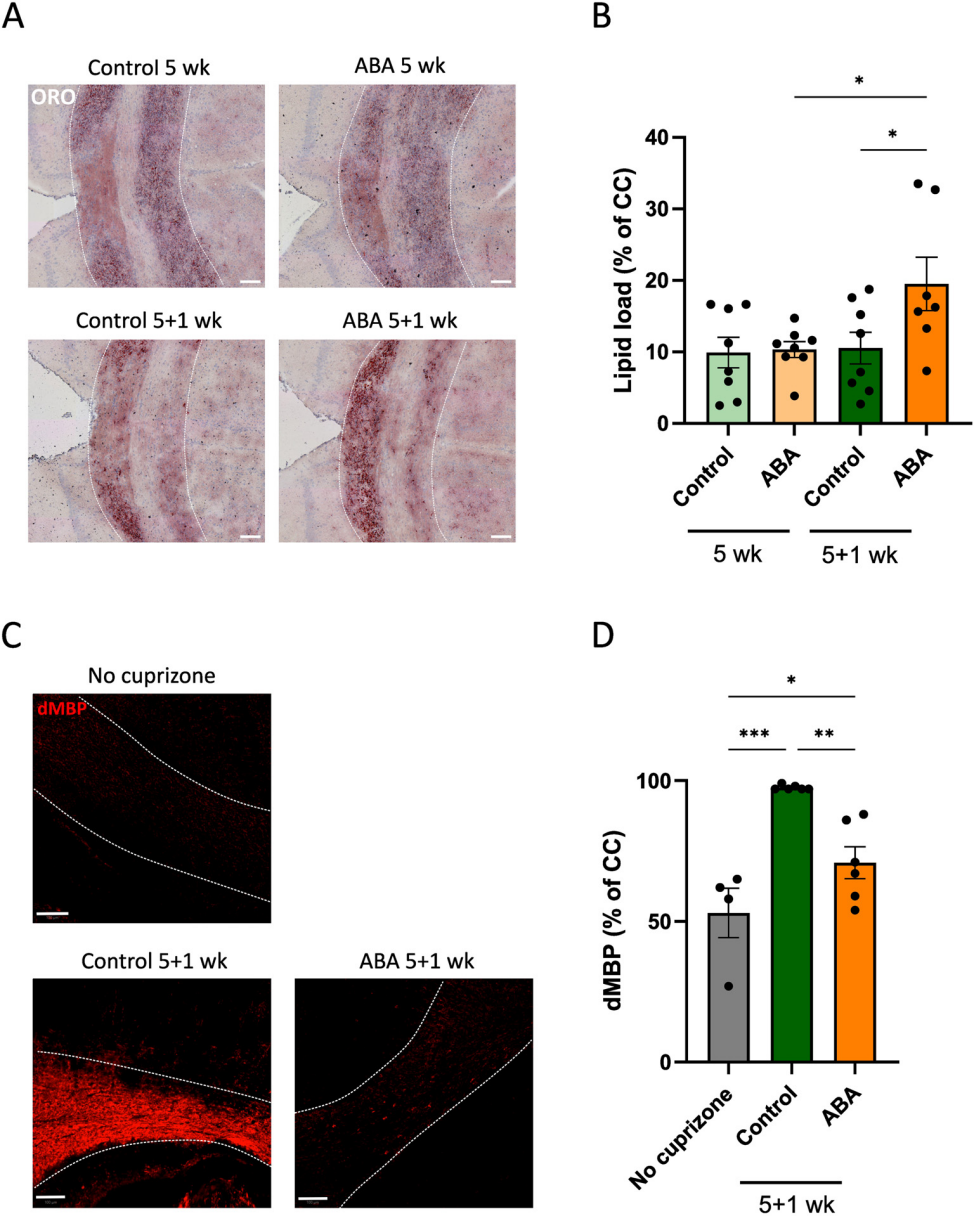
Impact of ABA on foam cell formation in the cuprizone model. **(A,C)** Representative images of ORO staining **(A)** and degraded MBP staining **(C)** of corpus callosum from mice fed with control or ABA-supplemented diet, after 5 weeks and 5 + 1 weeks. Scale bars, 100 µm. **(B, D)** Quantification of lipid load (defined as percent ORO+ area of total area of corpus callosum) **(B)** and degraded myelin (percent dMBP+ area of total corpus callosum in PFA-fixed non-permeabilized samples) **(D)** (n = 7-8 animals; 2-3 images per animal). Each dot represents one mouse. Data are represented as mean ± SEM and statistically analyzed using a one-way ANOVA with correction for multiple testing. *p<0.05, **p<0.01, ***p<0.001.

To investigate the impact of ABA on myelin clearance and processing by microglia, we initially examined foam cell formation. Counterintuitively, enhanced remyelination in ABA-fed mice was associated with increased intracellular lipid load, as evidenced by a higher ORO staining load (p<0.05) (**Figures 4A, B**). This observation prompted us to evaluate whether ABA facilitates remyelination by promoting the clearance of repair-inhibitory myelin debris (8). To this end, we quantified the presence of non-cell-associated myelin debris (31) by staining for MBP on PFA-fixed tissues without extensive permeabilization (72). Our results revealed a low MBP signal in both healthy mice and ABA-fed mice during remyelination, in contrast to a strong MBP signal in control-fed mice during remyelination (p<0.0002 and p<0.05 respectively) (**Figures 4C, D**). These findings indicate a higher presence of degraded myelin in control-fed mice compared to ABA-fed mice during remyelination, suggesting that the reparative impact of ABA is associated with increased myelin debris clearance and elevated intracellular lipid load.

**Figure 5:**
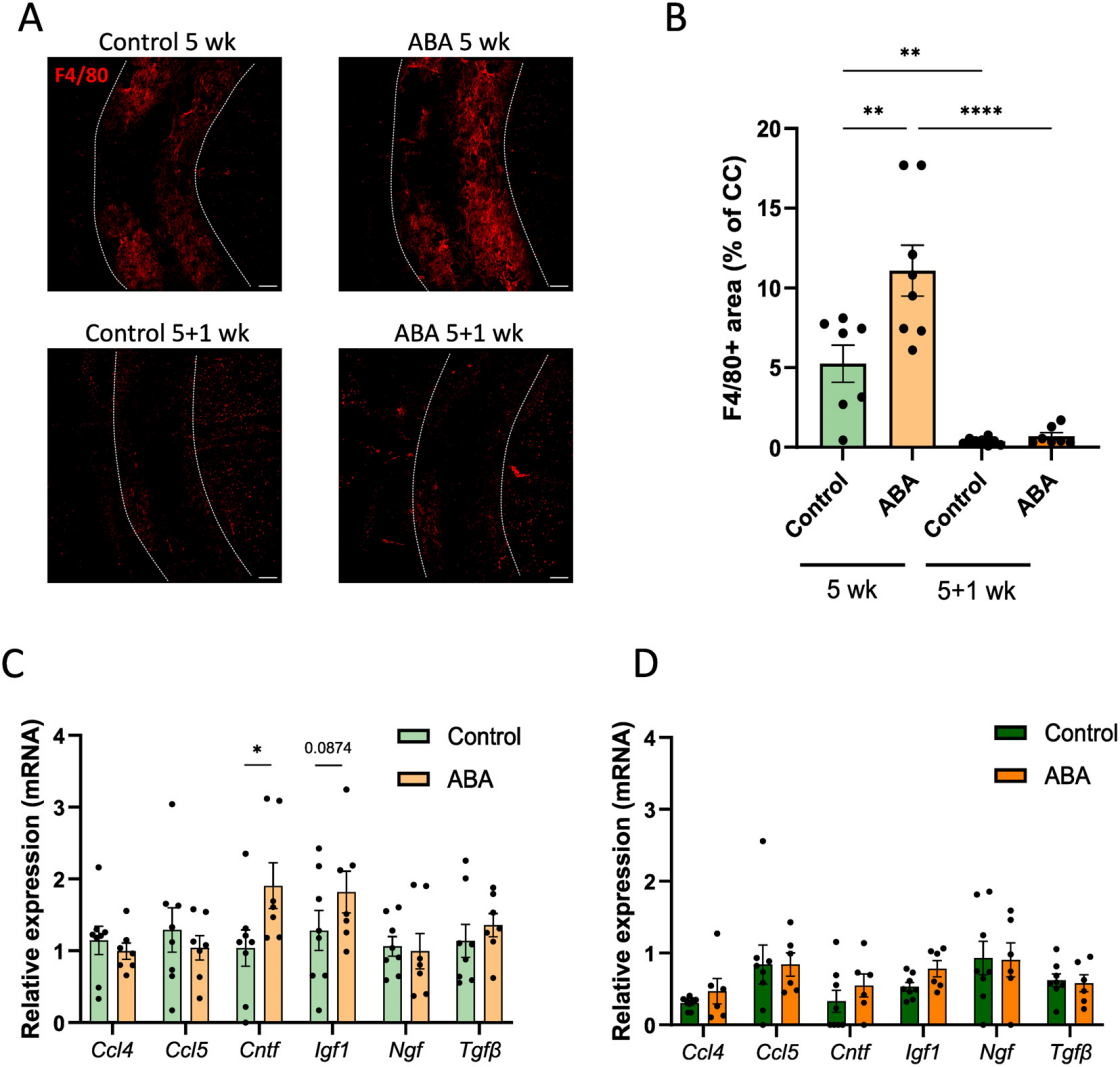
ABA affects phagocytes in corpus callosum from mice in the cuprizone model. **(A-B)** Representative immunofluorescence images **(A)** and quantification **(B)** of F4/80 staining of corpus callosum from mice fed with control or ABA-supplemented diet, after 5 weeks and 5 + 1 weeks. The outer border of the corpus callosum is demarcated by the dotted line. Scale bars, 200 µm. (n = 7-8 animals; 2 images per animal) **(C-D)** mRNA expression of *Ccl4, Ccl5, Cntf, Igf1, Ngf* and *Tgf*β in the corpus callosum from control-fed and ABA-fed mice after 5 weeks and 5 + 1 weeks (n = 7–8 animals). Each dot represents one mouse. All data are represented as mean ± SEM and statistically analyzed using a Student’s t-test. *p<0.05, **p<0.01, ****p<0.0001.

Furthermore, improved remyelination in ABA-fed mice was associated with a significantly higher abundance of F4/80+ phagocytes during the demyelination phase (p<0.002) (**Figures 5A, B**). However, ABA treatment did not affect the percentage of iNOS+ phagocytes (**Supplementary Figure S2**), nor did it result in significant changes in the mRNA expression of inflammatory mediators such as C-C motif chemokine 4 (*Ccl4)* and C-C motif chemokine 5 (*Ccl5)* (**Figures 5C, D**). Interestingly, ABA did affect the mRNA expression of neurotrophic factors. Specifically, ciliary neurotrophic factor (*Cntf)* was significantly increased during the demyelination phase (p<0.02), and there was a trend towards increased insulin growth factor 1 (*Igf1)* expression (p=0.0874). No significant differences were found for nerve growth factor (*Ngf)* or tumor growth factor β (*Tgf*β*)* (**Figures 5C, D**). Subsequently, we assessed whether microglia depletion using clodronate liposomes in lysolecithin-demyelinated cerebellar brain slices would counteract the protective effects of ABA. Our results showed that the absence of microglia abrogated the protective effects of ABA on remyelination (**Supplementary Figure S3**), indicating that ABA promotes remyelination in a microglia-dependent manner.

**Figure 6:**
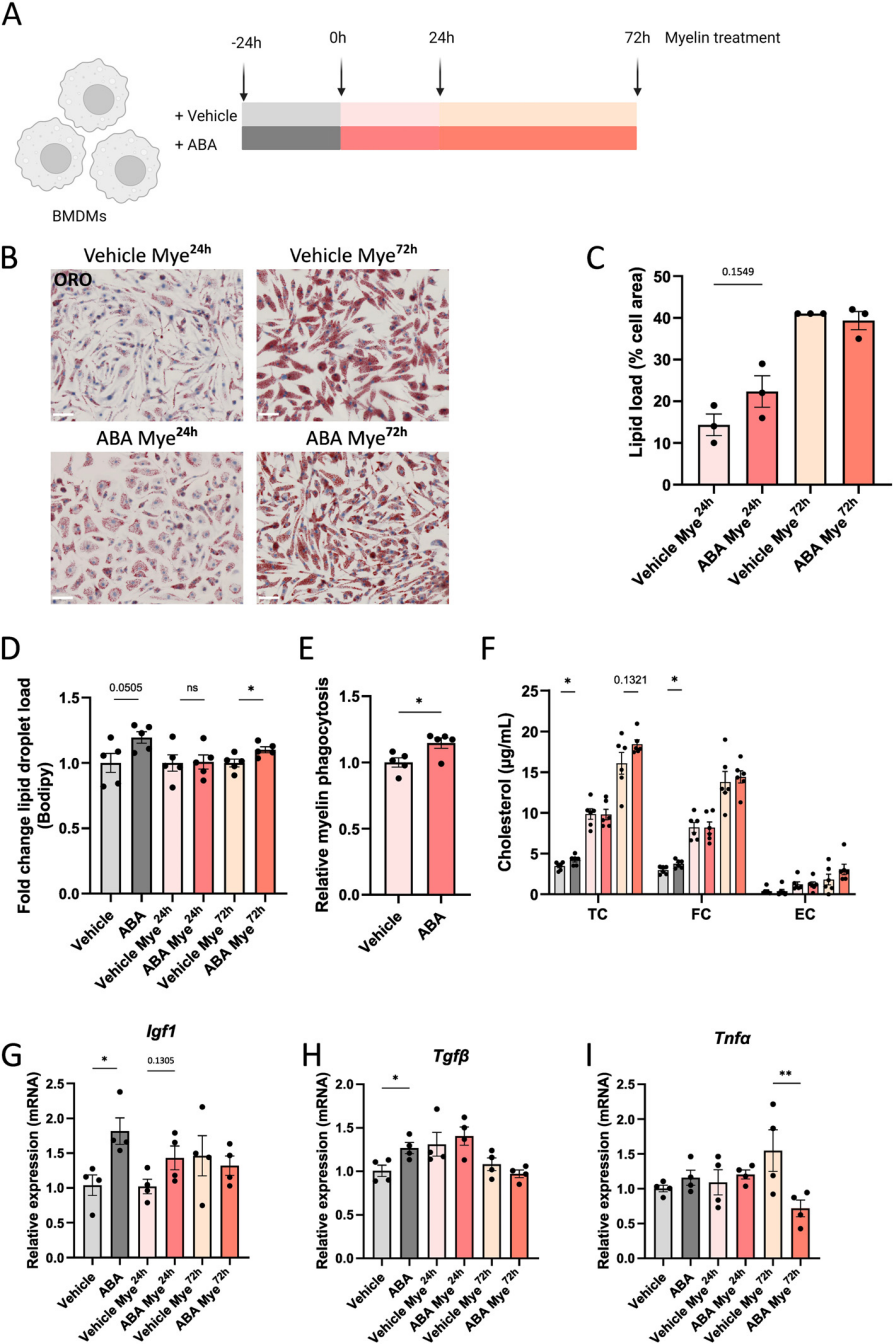
ABA promotes lipid droplet load in macrophages in vitro. **(A)** Schematic representation showing the experimental set-up: mouse BMDMs were left untreated or treated with 100µg/ml myelin for 24 hours (Mye^24h^) or 72 hours (Mye^72h^). Myelin exposure was performed in the presence or absence of ABA. Created with biorender.com. **(B)** Representative images of ORO staining of BMDMs exposed to vehicle (PBS) or ABA and treated with myelin for 24 or 72 hours. Scale bars, 50 µm. **(C)** Quantification of lipid load (defined as percent ORO+ area of total cell area)(n = 3). **(D)** Mean fluorescence intensity of BODIPY in BMDMs (n = 5) exposed to vehicle or ABA and treated with myelin for 24 or 72 hours, as measured by flow cytometry. Data is represented as relative lipid droplet load compared to vehicle. **(E)** Internalization of pHrodo™-labelled myelin by BMDMs exposed to vehicle or ABA for 24 hours. Data are measured by flow cytometry and depicted relative to the vehicle treated group. **(F)** Quantification of total cholesterol (TC), free cholesterol (FC), and esterified cholesterol (EC) in BMDMs (n = 6) exposed to vehicle or ABA and treated with myelin for 0, 24 or 72 hours. **(G-I)** mRNA expression of *Igf1, Tgfβ* and *Tnf*α in BMDMs (n = 4). Each dot represents one well. Data are represented as mean ± SEM and statistically analyzed using a one-way ANOVA with correction for multiple testing or Student’s t-test. *p<0.05, **p<0.01.

Given the critical role of F4/80+ cells in promoting remyelination, we were prompted to further investigate this cell population. For our *in vitro* experiments, we used primary BMDMs, as these cells are known to exhibit similar responses to myelin as microglia (11–13). BMDMs were cultured in the presence or absence of ABA and treated with myelin for 24 or 72 hours (experimental set-up in **Figure 6A**). Consistent with *in vivo* observations, there was a trend towards increased intracellular lipid levels in ABA-treated cells after 24 hours of myelin exposure (p=0.1549), as shown by ORO staining (**Figures 6B, C**). However, no differences were detected at the 72-hour time-point. Further analysis revealed an increase in lipid droplet load in ABA-treated cells at steady state (p=0.0505) and after 72 hours of myelin exposure (p<0.03) (**Figure 6D**). To determine whether increased lipid uptake underlies the observed increase in lipid droplets following ABA treatment, we assessed the capacity of BMDMs to phagocytose myelin. Phagocytosis experiments demonstrated an increased uptake of pHrodo™-labelled myelin by BMDMs exposed to ABA (p<0.03) (**Figure 6E**). Supporting these findings, total cholesterol (TC) and free cholesterol (FC) levels were significantly increased at steady state in the ABA group (p<0.04 and p<0.02 respectively), with a trend towards increased TC after 72 hours of myelin exposure (p=0.1321) (**Figure 6F**). These findings indicate that the changes in the cellular lipid droplet pool upon ABA exposure are likely due to an enhanced capacity to internalize extracellular lipid-containing ligands.

### 3.3 Abscisic acid has the capacity to execute anti-inflammatory effects

**Figure 7:**
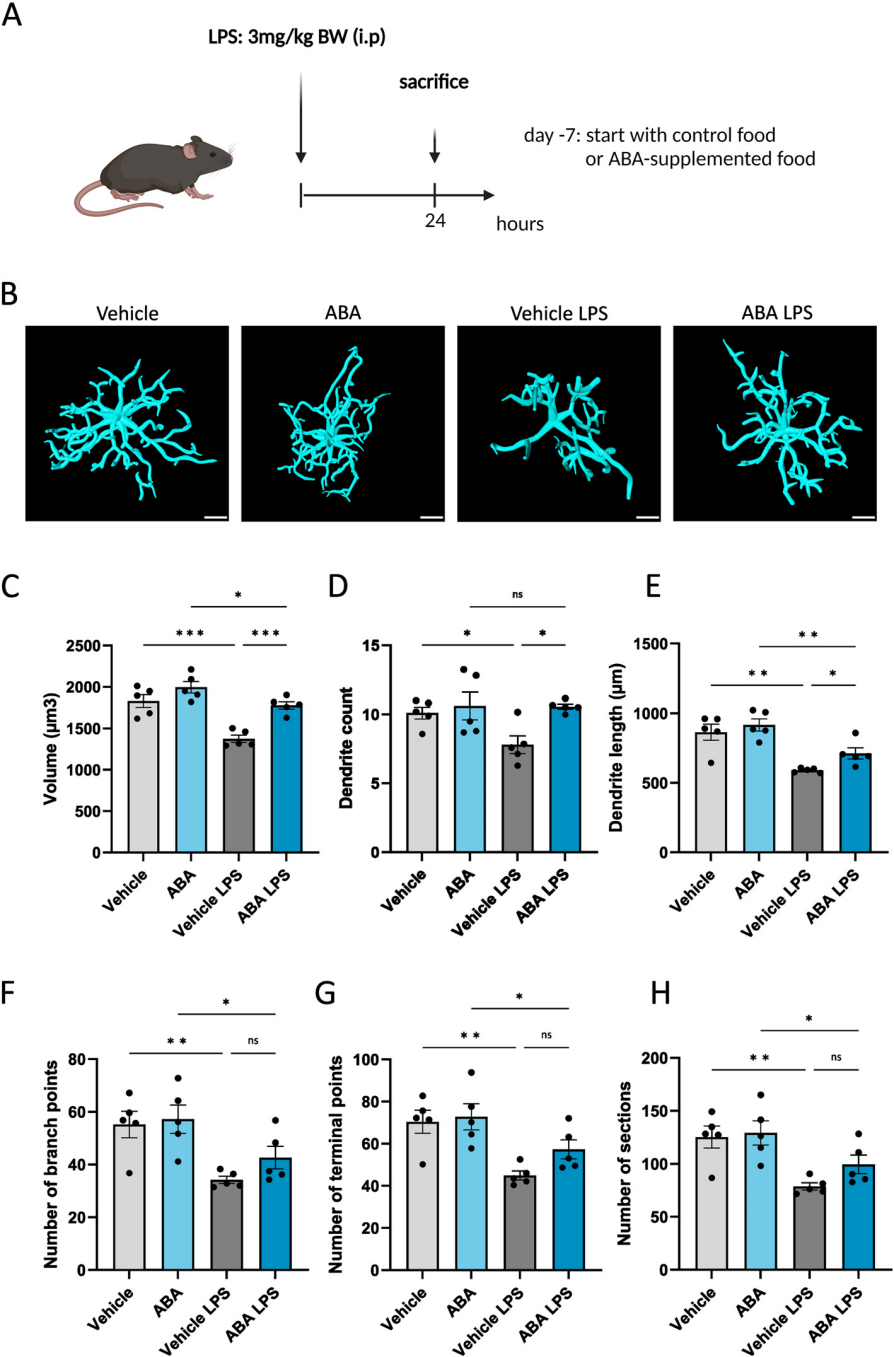
ABA affects microglial activation in LPS model. **(A)** Schematic representation of the experimental design: mice were pretreated with control or ABA-supplemented diet and injected interperitoneally with vehicle (PBS) or with LPS. Created with biorender.com. **(B)** Representative 3D reconstruction images of IBA1+ microglia in CA1 region of hippocampus 24 hours after Vehicle or LPS stimulation. Scale bars, 10 µm. **(C-H)** Arivis Vision 4D-based quantification of cell morphology of IBA1+ microglia in hippocampus. Each dot represents one mouse (n =5). For each mouse 4-10 cells were analyzed. Data are represented as mean ± SEM and statistically analyzed using a one-way ANOVA with correction for multiple testing. *p<0.05, **p<0.01, ***p<0.001.

Given that ABA has been reported to reduce inflammation in models of inflammatory bowel disease (47), Alzheimer’s disease (57) and HFD-induced neuroinflammation (56), we hypothesized that ABA could promote an anti-inflammatory and reparative phenotype in phagocytes. To investigate this, we used *in vitro* cultures of myelin-exposed phagocytes. We observed a significant increase in mRNA expression of neurotrophic factors *Igf1* and *Tgf*β at steady state (no myelin exposure) (p<0.008 and p<0.05 respectively), with a trend towards increased *Igf1* expression after 24 hours of myelin exposure (p=0.1305) (**Figures 6G, H**). This was consistent with the increased expression of *Cntf* and *Igf1* during the demyelination phase of the cuprizone experiment (**Figure 5C**). Although no differences were observed in the expression of inflammatory markers, including *Ccl4* and *Ccl5, in vivo* and *in vitro* (**Figure 5C**), there was a significant decrease in *Tnf*α expression after 72 hours of myelin exposure (p<0.002) (**Figure 6I**), suggesting a potential anti-inflammatory effect of ABA. To further explore this possibility, an *in vivo* LPS model was employed. Mice were fed either a control diet or an ABA-supplemented diet for one week, followed by a single LPS injection. The mice were evaluated 24 hours post-injection (experimental set-up in **Figure 7A**). To verify the effectiveness of diet and its ability to cross the blood-brain barrier, ABA levels were determined in both serum and whole brain samples, showing significant increases in ABA concentration (p<0.05) (**Supplementary Figure S4**). Upon LPS injection, microglia typically shift from a resting phenotype to an activated phenotype. 3D- modeling analysis was used to compare microglial phenotypes in the CA1 region of the hippocampus across experimental groups. LPS treatment resulted in a significant decrease in microglial volume (p<0.03) (**Figures 7B, C**). However, in ABA-fed mice, volume of microglia was significantly higher compared to control-fed mice (p<0.0005), indicating that microglia in the ABA group were less activated. Additionally, dendrite count and -length were significantly increased in the ABA-treated group (p<0.03 and p<0.04 respectively) (**Figures 7D, E**). No significant differences were detected in the number of branch points, terminal points, or sections in LPS-injected mice treated with vehicle or ABA (**Figures 7F–H**). These findings suggest a more resting, less inflammatory phenotype in ABA-treated mice. To further investigate this, inflammatory cytokines and chemokines were measured in serum, revealing a significant decrease in MCP 1 (p<0.006) (**Supplementary Figure S5**). These data collectively suggest that ABA may exert an anti-inflammatory effect by promoting a less activated state in microglia and by reducing pro-inflammatory chemokine levels. Despite the demonstrated protective effects of ABA in both the cuprizone-induced demyelination model (**Figure 2**) and the LPS-induced microglial activation model (**Figure 7**), we did not observe a protective effect on clinical score in the EAE model. Moreover, ABA-fed mice even exhibited a greater decrease in body weight during the acute phase of EAE (**Supplementary Figure S6**).

### 3.4 Abscisic acid promotes remyelination and lipid uptake in a PPARγ-dependent manner

**Figure 8:**
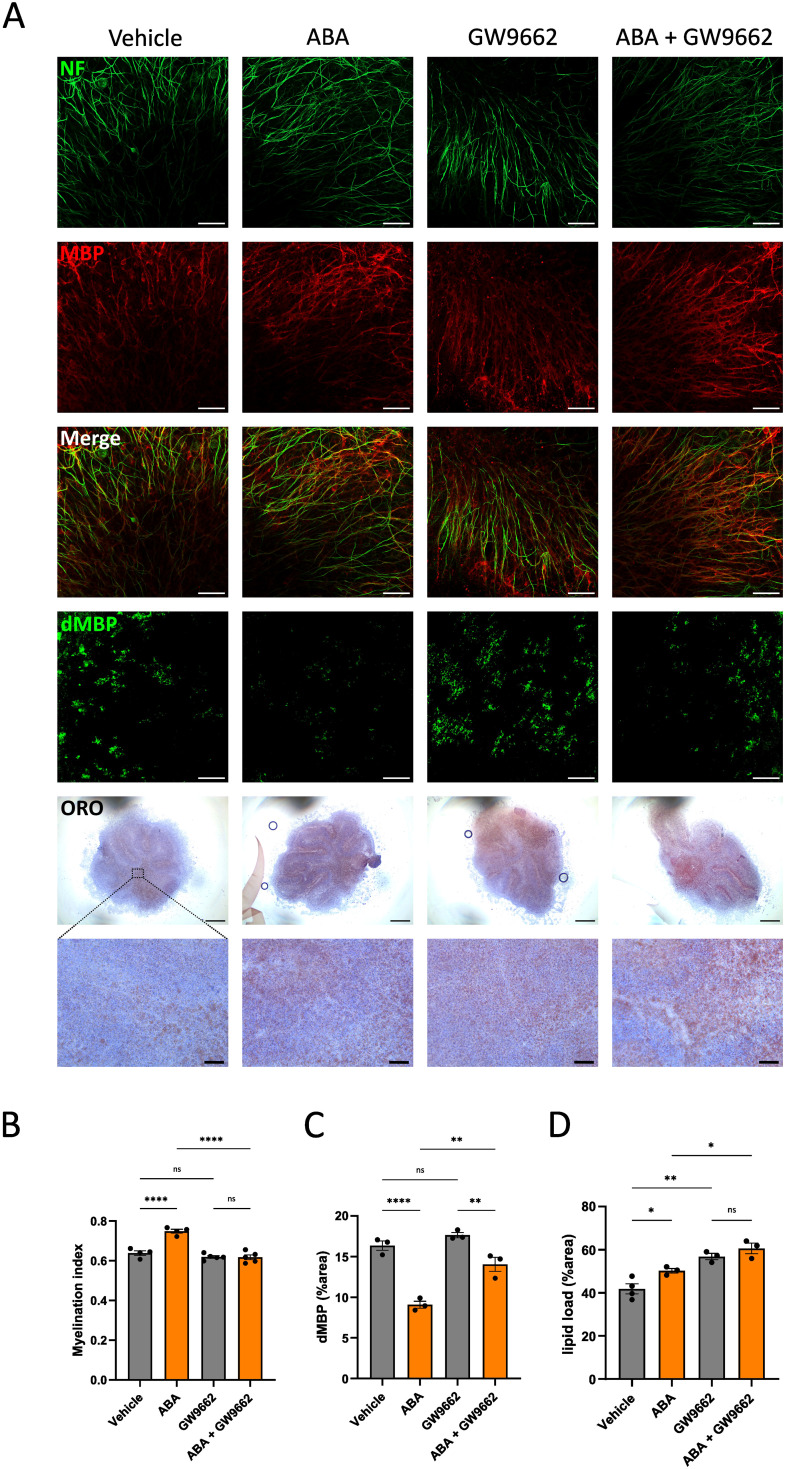
PPARγ affects remyelination and clearance of myelin debris. **(A)** Representative images of immunofluorescent MBP/NF, immunofluorescent dMBP and immunohistochemical ORO stains of cerebellar brain slices treated with vehicle or ABA in the presence or absence of PPARγ inhibitor (GW 9662). Scale bars, 50 μm (MBP/NF), 100 µm (dMBP), 500µm and 60µm (ORO). **(B-D)** Quantification of staining in cerebellar brain slices treated with vehicle or ABA in the presence or absence of PPARγ inhibitor (GW 9662). **(B)** Relative number of MBP+ NF+ axons out of total NF+ axons (n = 6). **(C)** Quantification of dMBP+ (defined as percent dMBP+ area of total area) (n = 4). **(D)** Quantification of lipid load (defined as percent ORO+ area of total area) (n = 3-4). Each dot represents one slice. Data are represented as mean ± SEM and statistically analyzed using a one-way ANOVA with correction for multiple testing. *p<0.05, **p<0.01, ****p<0.0001.

Our data indicated that ABA enhances lipid droplet formation by promoting the uptake of lipid-containing complexes by macrophages (**Figures 6B–F**). To identify the pathway underlying this increased phagocytic capacity following ABA exposure, we investigated PPARγ, a receptor frequently implicated as an important downstream signaling component for ABA and reported to function in a LANCL2-dependent manner (59, 73). In the cuprizone model, we observed a significant upregulation of *Lancl2* and *Pparγ* expression in the corpus callosum of ABA-fed mice during the remyelination phase (p<0.02 and p<0.04 respectively) (**Supplementary Figure S7A**). Additionally, the PPARγ response gene *Plin2* was significantly upregulated in ABA-fed mice during the demyelination phase (p<0.05), while no significant differences were detected in *Cd36* expression (**Supplementary Figure S7A**). Furthermore, we assessed the expression of *Lancl2*, *Pparγ*, and PPARγ-response genes *in vitro* using BMDMs exposed to myelin. Consistent with the *in vivo* results, ABA-treated BMDMs showed a significant increase in *Pparγ* expression at steady state (p<0.03) and a trend towards increased expression after 24 hours of myelin exposure (p=0.0861) (**Supplementary Figure S7B**). Additionally, expression of *Cd36* was also significantly increased at steady state in ABA-treated BMDMs (p<0.05). However, no differences were observed in the expression of *Lancl2* or *Plin2* (**Supplementary Figure S7B**). The pronounced increase in *Pparγ* expression in both ABA-treated mice during remyelination and BMDMs suggests that the effects of ABA on enhancing remyelination, increasing lipid droplet formation, and promoting uptake of myelin debris might be dependent on PPARγ. To determine whether PPARγ is involved in these effects, we conducted an *ex vivo* experiment using cerebellar brain slices demyelinated with lysolecithin and subsequently treated with ABA in the presence of a PPARγ inhibitor (GW9662). In this setting, the previously observed increase in the myelination index seen in ABA-treated slices was abolished when PPARγ was inhibited (**Figures 8A, B**). To further explore the underlying mechanism, we examined the impact of PPARγ inhibition on the ABA-induced enhancement of myelin clearance by phagocytes. Consistent with previous *in vivo* findings, ABA treatment in the *ex vivo* brain slice model resulted in a significant reduction in non-cell-associated myelin debris (p<0.0001) (**Figures 8A, C**). However, when PPARγ was inhibited, ABA-treated samples still exhibited a decrease in myelin debris, but the reduction was less pronounced compared to samples without the inhibitor (p<0.01) (**Figures 8A, C**). To determine if this effect was related to microglia-mediated uptake, we measured the intracellular lipid load. In line with prior *in vivo* and *in vitro* data, ABA treatments significantly increased intracellular lipid accumulation in the *ex vivo* model (p<0.04) (**Figures 8A, D**). Interestingly, the PPARγ inhibitor alone also significantly elevated lipid load (p<0.002), with no additional increase observed in the presence of ABA (**Figures 8A, D**). These findings suggest that ABA might enhance remyelination through a PPARγ-dependent mechanism, primarily by facilitating the clearance of myelin debris.

## 4 Discussion

Over the past few decades, there has been a sudden rise in the incidence of MS in Western countries (39). However, many of the drugs currently used to treat neurological conditions often prove ineffective for certain patients and can cause adverse effects (39). This highlights a significant gap in the current therapies, necessitating the identification of safer and more efficient treatments. Epidemiological studies have demonstrated that environmental factors play a more substantial role in the development of MS than genetic factors (40, 74, 75). Diet in particular is a well-known contributor to MS (41, 42). Specifically, increased sodium intake and excessive fat consumption are associated with heightened disease exacerbations and more severe EAE (76, 77). Despite the detrimental effects of a Western diet, it also contains small amounts of components that counteract disease-promoting compounds. Flavonoids, such as quercetin and epigallocatechin gallate, are known to reduce the production of pro-inflammatory cytokines by macrophages and microglia (78, 79). While these flavonoids may have some capacity to modulate disease mechanisms in MS, other plant-derived compounds may have a higher potential to influence MS pathogenesis. Compounds abundant in the Mediterranean diet are often regarded as promising candidates (80). Therefore, in this study, we investigated ABA, which is prevalent in the Mediterranean diet and in fruits such as figs, apricots and bilberries. We provide evidence that ABA promotes remyelination *ex vivo* and *in vivo.* Enhanced remyelination is evidenced by increased MBP staining and a higher number of myelinated axons with higher g-Ratio seen on TEM. These findings indicate that ABA may be useful for promoting the repair of damage within the CNS.

We investigated the mechanism of enhanced remyelination using an *in vitro* model that simulates the formation of foamy macrophages and microglia in the CNS, and found that ABA treatment leads to elevated intracellular lipid levels. Specifically, we demonstrate that the changes in the cellular lipid droplet pool following ABA exposure are likely due to an enhanced capacity to internalize extracellular lipid-containing ligands. Furthermore, validations using *ex vivo* and *in vivo* models further substantiated these findings, confirming that ABA exposure is associated with increased cholesterol accumulation in phagocytes. Since increased cholesterol accumulation in phagocytes has been linked to both enhanced (12, 36) and reduced (81) remyelination capacity, further investigation into the underlying mechanisms was warranted. Additional measurements revealed reduced levels of degraded myelin upon ABA treatment, suggesting that improved clearance of myelin debris may be driving the enhanced lipid load.

Furthermore, our findings reveal that ABA exposure not only increases lipid droplet load but is also associated with a significantly higher abundance of F4/80^+^ phagocytes during demyelination. While phagocytes can be both disease-promoting and disease-resolving (12, 21–26), our *in vitro* studies of ABA-treated foamy phagocytes reveal a significant decrease in *Tnfα* expression, suggesting a potential anti-inflammatory effect of ABA. To further investigate this potential, an *in vivo* low-dose LPS model was employed. LPS models, similar to most neurological disorders, are characterized by microglial activation (82, 83). This activation involves significant morphological and transcriptional alterations in microglia, which aim to mitigate CNS damage and facilitate repair. However, this process frequently leads to excessive inflammation, thereby perpetuating and exacerbating neurodegenerative progression. At steady state, microglia typically exhibit a resting phenotype, actively surveying the brain. Upon encountering toxic stimuli such as injury, infection, dead cells, or misfolded proteins, microglia become activated, characterized by a progressive loss of branches and a more rounded shape. Previous studies have demonstrated that ABA can revert microglia to a less activated state in models of HFD-induced neuroinflammation (55, 56) and Alzheimer’s disease (57, 60). Additionally, Bassaganya-Riera et al. showed that ABA reduces proinflammatory cytokine production in LPS-induced murine sepsis models (61). LPS injection in mice typically causes microglia to shift from a resting phenotype to an activated one. However, in our study, mice pre-treated with ABA maintained a more resting, less inflammatory microglial phenotype compared to control-fed mice and exhibited reduced expression of MCP1. These findings suggest that ABA may exert an anti-inflammatory effect by promoting a less activated state in microglia and reducing pro-inflammatory chemokine levels. These results align with previous reports by Maixner et al., which showed that ABA treatment reduced TNFα production and decreased Iba1 protein expression, a marker of microglial activation (69). Nonetheless, it remains unclear whether ABA has a direct effect on microglial polarization or if it for example reduces the toxic burden in neurons, thereby diminishing the toxic signals to microglia (60).

Although we demonstrated that ABA has protective effects in both the cuprizone-induced demyelination model and the LPS-induced microglial activation model, no protective effects were observed in the EAE model. In fact, ABA-fed mice exhibited a greater decrease in body weight during the acute phase of EAE. Previous studies have reported that in EAE, persistent and pronounced microglial activation plays a detrimental role in CNS autoimmunity and that preventing or suppressing this activation may have therapeutic benefits (83). A seminal study by Heppner et al. provided the first direct evidence that “microglial paralysis”, characterized by microglia with reduced capacity to proliferate, migrate and produce cytokines, leads to EAE suppression (84). However, the observed reduction in microglial activation in the LPS model did not translate to a protective effect on disease progression in EAE. This discrepancy can be explained by the fact that, in the EAE model, demyelination is dependent on and accompanied by a T cell-mediated immune response (85), which may act as a confounding factor. In our experiment, ABA-treatment was started before EAE induction and may therefore lead to more efficient uptake of the EAE induction agent by dendritic cells and thereby enhance T cell responses. We suggest that treatment with ABA during the chronic phase of EAE may reverse the course of disease or improve recovery of EAE and should be investigated. However, the cuprizone model remains the most straightforward for studying microglial-dependent innate immune mechanisms and exploring strategies to directly affect oligodendrocyte survival and differentiation, as well as to promote remyelination. Nonetheless, given that ABA can impact a variety of cell types, we cannot exclude the possibility that its protective effects may involve mechanisms other than those described above.

In this study, we put forward PPARγ as the receptor driving the increased remyelinating capacity observed upon ABA exposure. PPARγ, which is expressed in a multitude of tissues (86) including adipose tissue (58, 87), intestinal epithelial cells (88), lymphocytes, macrophages, neurons, microglia, astrocytes and oligodendrocytes (86, 89), is frequently implicated as an important downstream signaling component for ABA (59). Both ABA and PPARγ agonists have previously been reported to ameliorate memory performance in Alzheimer’s disease (59, 90, 91) and dietary ABA has been shown to upregulate PPARγ in immune cells and thereby reduce the severity of inflammatory bowel disease and type 2 diabetes in mice (47, 58). In the cuprizone-induced model for MS, we show that ABA treatment is associated with significantly increased expression of *Pparγ* during the recovery phase. Since previous studies have demonstrated that PPARγ and PPARγ-response genes such as *Plin2* and *Cd36* are upregulated by myelin internalization (38, 92, 93), the observed upregulation of *Plin2* in ABA-treated mice during the demyelination phase suggests that ABA promotes lipid uptake. Supporting this finding, we observed increased expression of *Cd36*, which encodes a phagocytic receptor involved in myelin uptake (93), in ABA-treated BMDMs at steady state. Using an *ex vivo* model where cerebellar brain slices were treated with ABA in the presence of a PPARγ inhibitor (GW9662), we showed that PPARγ plays a critical role in ABA-dependent remyelination, lipid uptake and clearance of myelin debris. However, interpretation of the results was complicated by the observation that lipid load was already significantly elevated in brain slices treated with the PPARγ inhibitor alone, an effect thought to result from impaired intracellular lipid processing rather than increased phagocytosis (38). This complicated interpretation combined with the observation of increased *Cd36* expression in ABA-treated resting BMDMs, but not in myelin-exposed BMDMs or cuprizone-treated mice, suggests that additional research is necessary to fully understand the PPARγ-mediated effects induced by ABA. Investigating the effects of ABA on remyelination and clearance of myelin debris in PPARγ knockout mouse models may provide clearer insight into the role of PPARγ. Although our findings suggest that the reparative impact of ABA relies on PPARγ activation, it was previously reported that ABA does not bind to the ligand-binding-domain of PPARγ (61) and expression of PPARγ in intestinal epithelial cells was not required for the anti-inflammatory efficacy of ABA in inflammatory bowel disease (88). Furthermore, it was demonstrated that ABA binds to LANCL2 and that the ABA/LANCL2 system regulates inflammation signaling pathways in mammalian cells or tissues (51, 62). The exact mechanism by which ABA indirectly activates PPARγ remains to be elucidated. Here, we show that in addition to *Pparγ*, *Lancl2* expression is also increased in the corpus callosum of ABA-fed mice during the recovery phase of the cuprizone model, suggesting an important role for the PPARγ/LANCL2 axis in regulating the protective effects of ABA.

In summary, we indicate that ABA enhances remyelination by stimulating myelin uptake and clearance of myelin debris by macrophages. The anti-inflammatory effects of ABA in the LPS model, combined with its capacity to improve remyelination in a cuprizone model, make ABA a promising compound to modulate macrophage phenotype and neuroinflammation in MS. However, it is important to note that our data are based on *in vitro* BMDM models, *ex vivo* brain slice cultures and mouse models. While these experimental models are considered the gold standard for studying CNS regeneration, human remyelination may not precisely replicate the pathological and regenerative changes observed in these models. Additionally, some studies suggest that mice exhibit notable differences in lipid metabolism compared to humans, highlighting the need for caution when translating these findings to clinical settings.

## Data Availability

The original contributions presented in the study are included in the article/**Supplementary Material**. Further inquiries can be directed to the corresponding author/s.

## Supplementary material

Supplementary Figure 1 ABA is increased in serum from ABA-fed mice. **(A)** ABA measurement in serum from mice given a cuprizone supplemented control or ABA diet (400 mg/kg). Serum was taken at sacrifice, after 5 weeks for the demyelination group and after 5 + 1 weeks for the remyelination group. ABA is measured by PYL1H87P-based biosensor. Each dot represents one mouse (n = 7-8). Data are represented as mean ± SEM and statistically analyzed using a Student’s t-test. **p<0.01, ***p<0.001.

Supplementary Figure 2 Measurement of iNOS staining. **(A)** Representative images of immunofluorescent iNos staining of the corpus callosum from mice fed with control diet or ABA-supplemented diet. The outer border of the corpus callosum is demarcated by the dotted line. Scale bars, 200 μm. **(B)** Quantification of the percentage iNos+ F4/80+ area out of total F4/80+ area in the corpus callosum from cuprizone animals fed with control diet or ABA diet (n = 6-8 animals; 2 images per animal). Each dot represents one mouse. Data are represented as mean ± SEM and statistically analyzed using a Student’s t-test.

Supplementary Figure 3 Measurement of remyelination efficiency in microglia-depleted brain slice cultures. **(A)** Representative images of immunofluorescent MBP/NF stains of cerebellar brain slices treated with vehicle or ABA, without lipsomes, with empty liposomes (control liposomes) or clodronate liposomes. Scale bars, 50 μm. **(B)** Relative number of MBP+ NF+ axons out of total NF+ axons in cerebellar brain slices stimulated with control liposomes or clodronate liposomes (0.5 mg/mL) and treated with vehicle or ABA (n = 3 slices). Each dot represents one slice. Data are represented as mean ± SEM and statistically analyzed using a Student’s t-test. *p<0.05, **p<0.01.

Supplementary Figure 4 Measurement of ABA in serum and brain lysates from mice in the LPS model. **(A-B)** ABA measurement in serum **(A)** and brain lysate **(B)** from mice fed a control or ABA-supplemented diet and subjected to LPS injection. ABA is measured by PYL1H87P-based biosensor. Each dot represents one mouse. Data are represented as mean ± SEM and statistically analyzed using a Student’s t-test. *p<0.05, **p<0.01.

Supplementary Figure 5 Cytokine and chemokine measurements in serum from mice in the LPS model. **(A-F)** Protein levels of IL6 **(A)**, MCP1 **(B)**, IFNγ **(C)**, IL10 **(D)**, CCL5 **(E)**, and TNFα **(F)** in plasma as measured by Bioplex (n = 5). Each dot represents one mouse (n = 5). Data are represented as mean ± SEM and statistically analyzed using a one-way ANOVA with correction for multiple testing. *p < 0.05, **p < 0.01, ***p<0.001, ****p<0.0001.

Supplementary Figure 6 ABA has no effect on disease progression in an experimental autoimmune encephalomyelitis (EAE) model. **(A)** Schematic representation of the experimental design: mice were treated with control or ABA-supplemented diet starting 1 week before EAE induction and continued until the end of the experiment. From the day of immunization, daily measurements of body weight and clinical disease symptoms was performed. Created with biorender.com. **(B, C)** Body weight (g) measurements **(B)** and clinical disease scores **(C)** of ABA treated EAE mice versus control EAE mice (n = 8).

Supplementary Figure 7 ABA affects mRNA expression of *Lancl2*, *Pparγ*, *Cd36* and *Plin2*. **(A)** mRNA expression of *Lancl2*, *Pparγ, Cd36* and *Plin2* in the corpus callosum from control-fed and ABA-fed mice after 5 weeks and 5 + 1 weeks of cuprizone treatment (n = 7–8 animals). Each dot represents one mouse. One outlier was removed for *Plin2* after ROUT method of detecting outliers with Q = 0.1% (*Plin2:* n = 7). **(B)** mRNA expression of *Lancl2*, *Pparγ, Cd36* and *Plin2* in BMDMs (n = 5) exposed to vehicle or ABA and treated with myelin for 0, 24 or 72 hours. Each dot represents one well. Data are represented as mean ± SEM and statistically analyzed using a Student’s t-test. *p<0.05.

## References

[1] Walton C King R Rechtman L Kaye W Leray E Marrie RA. Rising prevalence of multiple sclerosis worldwide: Insights from the Atlas of MS, third edition. Mult Scler Houndmills Basingstoke Engl. (2020) 26:1816–21. doi: 10.1177/1352458520970841 PMC7720355 33174475

[2] Franklin RJM Ffrench-Constant C. Regenerating CNS myelin - from mechanisms to experimental medicines. Nat Rev Neurosci. (2017) 18:753–69. doi: 10.1038/nrn.2017.136 29142295

[3] Filippi M Bar-Or A Piehl F Preziosa P Solari A Vukusic S. Multiple sclerosis. Nat Rev Dis Primer. (2018) 4:43. doi: 10.1038/s41572-018-0041-4 30410033

[4] Tallantyre EC Bø L Al-Rawashdeh O Owens T Polman CH Lowe JS. Clinico-pathological evidence that axonal loss underlies disability in progressive multiple sclerosis. Mult Scler Houndmills Basingstoke Engl. (2010) 16:406–11. doi: 10.1177/1352458510364992 20215480

[5] Stangel M Kuhlmann T Matthews PM Kilpatrick TJ. Achievements and obstacles of remyelinating therapies in multiple sclerosis. Nat Rev Neurol. (2017) 13:742–54. doi: 10.1038/nrneurol.2017.139 29146953

[6] Dillenburg A Ireland G Holloway RK Davies CL Evans FL Swire M. Activin receptors regulate the oligodendrocyte lineage in health and disease. Acta Neuropathol (Berl). (2018) 135:887–906. doi: 10.1007/s00401-018-1813-3 29397421 PMC5954071

[7] Keough MB Rogers JA Zhang P Jensen SK Stephenson EL Chen T. An inhibitor of chondroitin sulfate proteoglycan synthesis promotes central nervous system remyelination. Nat Commun. (2016) 7:11312. doi: 10.1038/ncomms11312 27115988 PMC4853428

[8] Lampron A Larochelle A Laflamme N Préfontaine P Plante MM Sánchez MG. Inefficient clearance of myelin debris by microglia impairs remyelinating processes. J Exp Med. (2015) 212:481–95. doi: 10.1084/jem.20141656 PMC4387282 25779633

[9] Fancy SPJ Kotter MR Harrington EP Huang JK Zhao C Rowitch DH. Overcoming remyelination failure in multiple sclerosis and other myelin disorders. Exp Neurol. (2010) 225:18–23. doi: 10.1016/j.expneurol.2009.12.020 20044992

[10] Franklin RJM. Why does remyelination fail in multiple sclerosis? Nat Rev Neurosci. (2002) 3:705–14. doi: 10.1038/nrn917 12209119

[11] Cantuti-Castelvetri L Fitzner D Bosch-Queralt M Weil MT Su M Sen P. Defective cholesterol clearance limits remyelination in the aged central nervous system. Science. (2018) 359:684–8. doi: 10.1126/science.aan4183 29301957

[12] Bogie JFJ Grajchen E Wouters E Corrales AG Dierckx T Vanherle S. Stearoyl-CoA desaturase-1 impairs the reparative properties of macrophages and microglia in the brain. J Exp Med. (2020) 217:e20191660. doi: 10.1084/jem.20191660 32097464 PMC7201924

[13] Marschallinger J Iram T Zardeneta M Lee SE Lehallier B Haney MS. Lipid-droplet-accumulating microglia represent a dysfunctional and proinflammatory state in the aging brain. Nat Neurosci. (2020) 23:194–208. doi: 10.1038/s41593-019-0566-1 31959936 PMC7595134

[14] Bogie JFJ Stinissen P Hendriks JJA. Macrophage subsets and microglia in multiple sclerosis. Acta Neuropathol (Berl). (2014) 128:191–213. doi: 10.1007/s00401-014-1310-2 24952885

[15] Prineas JW Parratt JDE. Multiple sclerosis: microglia, monocytes, and macrophage-mediated demyelination. J Neuropathol Exp Neurol. (2021) 80:975–96. doi: 10.1093/jnen/nlab083 PMC8557350 34553215

[16] Vogel DYS Vereyken EJF Glim JE Heijnen PDAM Moeton M van der Valk P. Macrophages in inflammatory multiple sclerosis lesions have an intermediate activation status. J Neuroinflammation. (2013) 10:35. doi: 10.1186/1742-2094-10-35 23452918 PMC3610294

[17] Zeng Y Peng Y Tang K Wang YQ Zhao ZY Wei XY. Dihydromyricetin ameliorates foam cell formation via LXRα-ABCA1/ABCG1-dependent cholesterol efflux in macrophages. BioMed Pharmacother Biomedecine Pharmacother. (2018) 101:543–52. doi: 10.1016/j.biopha.2018.02.124 29505925

[18] Xia M Hou M Zhu H Ma J Tang Z Wang Q. Anthocyanins induce cholesterol efflux from mouse peritoneal macrophages: the role of the peroxisome proliferator-activated receptor {gamma}-liver X receptor {alpha}-ABCA1 pathway. J Biol Chem. (2005) 280:36792–801. doi: 10.1074/jbc.M505047200 16107338

[19] Chang YC Lee TS Chiang AN. Quercetin enhances ABCA1 expression and cholesterol efflux through a p38-dependent pathway in macrophages. J Lipid Res. (2012) 53:1840–50. doi: 10.1194/jlr.M024471 PMC3413225 22711909

[20] Grajchen E Hendriks JJA Bogie JFJ. The physiology of foamy phagocytes in multiple sclerosis. Acta Neuropathol Commun. (2018) 6:124. doi: 10.1186/s40478-018-0628-8 30454040 PMC6240956

[21] Bogie JFJ Jorissen W Mailleux J Nijland PG Zelcer N Vanmierlo T. Myelin alters the inflammatory phenotype of macrophages by activating PPARs. Acta Neuropathol Commun. (2013) 2:43. doi: 10.1186/2051-5960-1-43 PMC3893408 24252308

[22] Bogie JFJ Timmermans S Huynh-Thu VA Irrthum A Smeets HJM Gustafsson JÅ. Myelin-derived lipids modulate macrophage activity by liver X receptor activation. PloS One. (2012) 7:e44998. doi: 10.1371/journal.pone.0044998 22984598 PMC3440367

[23] Boven LA Van Meurs M Van Zwam M Wierenga-Wolf A Hintzen RQ Boot RG. Myelin-laden macrophages are anti-inflammatory, consistent with foam cells in multiple sclerosis. Brain J Neurol. (2006) 129:517–26. doi: 10.1093/brain/awh707 16364958

[24] Koch-Henriksen N Sørensen PS. The changing demographic pattern of multiple sclerosis epidemiology. Lancet Neurol. (2010) 9:520–32. doi: 10.1016/S1474-4422(10)70064-8 20398859

[25] Berghoff SA Spieth L Sun T Hosang L Schlaphoff L Depp C. Microglia facilitate repair of demyelinated lesions via post-squalene sterol synthesis. Nat Neurosci. (2021) 24:47–60. doi: 10.1038/s41593-020-00757-6 33349711 PMC7116742

[26] Ruckh JM Zhao JW Shadrach JL van Wijngaarden P Rao TN Wagers AJ. Rejuvenation of regeneration in the aging central nervous system. Cell Stem Cell. (2012) 10:96–103. doi: 10.1016/j.stem.2011.11.019 22226359 PMC3714794

[27] Bogie JFJ Stinissen P Hellings N Hendriks JJA. Myelin-phagocytosing macrophages modulate autoreactive T cell proliferation. J Neuroinflammation. (2011) 8:85. doi: 10.1186/1742-2094-8-85 21781347 PMC3149992

[28] Hikawa N Takenaka T. Myelin-stimulated macrophages release neurotrophic factors for adult dorsal root ganglion neurons in culture. Cell Mol Neurobiol. (1996) 16:517–28. doi: 10.1007/BF02150231 PMC11563098 8879753

[29] Absinta M Sati P Masuzzo F Nair G Sethi V Kolb H. Association of chronic active multiple sclerosis lesions with disability *in vivo* . JAMA Neurol. (2019) 76:1474–83. doi: 10.1001/jamaneurol.2019.2399 PMC6692692 31403674

[30] Franklin RJM Ffrench-Constant C. Remyelination in the CNS: from biology to therapy. Nat Rev Neurosci. (2008) 9:839–55. doi: 10.1038/nrn2480 18931697

[31] Grajchen E Wouters E van de Haterd B Haidar M Hardonnière K Dierckx T. CD36-mediated uptake of myelin debris by macrophages and microglia reduces neuroinflammation. J Neuroinflammation. (2020) 17:224. doi: 10.1186/s12974-020-01899-x 32718316 PMC7384221

[32] Kotter MR Setzu A Sim FJ Van Rooijen N Franklin RJ. Macrophage depletion impairs oligodendrocyte remyelination following lysolecithin-induced demyelination. Glia. (2001) 35:204–12. doi: 10.1002/glia.v35:3 11494411

[33] Kotter MR Zhao C van Rooijen N Franklin RJM. Macrophage-depletion induced impairment of experimental CNS remyelination is associated with a reduced oligodendrocyte progenitor cell response and altered growth factor expression. Neurobiol Dis. (2005) 18:166–75. doi: 10.1016/j.nbd.2004.09.019 15649707

[34] McNamara NB Munro DAD Bestard-Cuche N Uyeda A Bogie JFJ Hoffmann A. Microglia regulate central nervous system myelin growth and integrity. Nature. (2023) 613:120–9. doi: 10.1038/s41586-022-05534-y PMC9812791 36517604

[35] Sariol A Mackin S Allred MG Ma C Zhou Y Zhang Q. Microglia depletion exacerbates demyelination and impairs remyelination in a neurotropic coronavirus infection. Proc Natl Acad Sci U S A. (2020) 117:24464–74. doi: 10.1073/pnas.2007814117 PMC7533697 32929007

[36] Vanherle S Jorissen W Dierckx T Loix M Grajchen E Mingneau F. The ApoA-I mimetic peptide 5A enhances remyelination by promoting clearance and degradation of myelin debris. Cell Rep. (2022) 41:111591. doi: 10.1016/j.celrep.2022.111591 36351388

[37] Mailleux J Vanmierlo T Bogie JF Wouters E Lütjohann D Hendriks JJ. Active liver X receptor signaling in phagocytes in multiple sclerosis lesions. Mult Scler Houndmills Basingstoke Engl. (2018) 24:279–89. doi: 10.1177/1352458517696595 28273782

[38] Wouters E Grajchen E Jorissen W Dierckx T Wetzels S Loix M. Altered PPARγ Expression promotes myelin-induced foam cell formation in macrophages in multiple sclerosis. Int J Mol Sci. (2020) 21:9329. doi: 10.3390/ijms21239329 33297574 PMC7731422

[39] Matveeva O Bogie JFJ Hendriks JJA Linker RA Haghikia A Kleinewietfeld M. Western lifestyle and immunopathology of multiple sclerosis. Ann N Y Acad Sci. (2018) 1417:71–86. doi: 10.1111/nyas.2018.1417.issue-1 29377214 PMC5947729

[40] Jörg S Grohme DA Erzler M Binsfeld M Haghikia A Müller DN. Environmental factors in autoimmune diseases and their role in multiple sclerosis. Cell Mol Life Sci CMLS. (2016) 73:4611–22. doi: 10.1007/s00018-016-2311-1 PMC5097114 27491297

[41] Thorburn AN Macia L Mackay CR. Diet, metabolites, and “western-lifestyle” inflammatory diseases. Immunity. (2014) 40:833–42. doi: 10.1016/j.immuni.2014.05.014 24950203

[42] Odegaard AO Koh WP Yuan JM Gross MD Pereira MA. Western-style fast food intake and cardiometabolic risk in an Eastern country. Circulation. (2012) 126:182–8. doi: 10.1161/CIRCULATIONAHA.111.084004 PMC4059207 22753304

[43] Yahfoufi N Alsadi N Jambi M Matar C. The immunomodulatory and anti-inflammatory role of polyphenols. Nutrients. (2018) 10:1618. doi: 10.3390/nu10111618 30400131 PMC6266803

[44] Prieto-Domínguez N Garcia-Mediavilla MV Sanchez-Campos S Mauriz JL Gonzalez-Gallego J. Autophagy as a molecular target of flavonoids underlying their protective effects in human disease. Curr Med Chem. (2018) 25:814–38. doi: 10.2174/0929867324666170918125155 28925866

[45] Kong Y Feng Z Chen A Qi Q Han M Wang S. The natural flavonoid galangin elicits apoptosis, pyroptosis, and autophagy in glioblastoma. Front Oncol. (2019) 9:942. doi: 10.3389/fonc.2019.00942 31612107 PMC6776614

[46] Kim SW Goossens A Libert C Van Immerseel F Staal J Beyaert R. Phytohormones: Multifunctional nutraceuticals against metabolic syndrome and comorbid diseases. Biochem Pharmacol. (2020) 175:113866. doi: 10.1016/j.bcp.2020.113866 32088261

[47] Guri AJ Evans NP Hontecillas R Bassaganya-Riera J. T cell PPARγ is required for the anti-inflammatory efficacy of abscisic acid against experimental IBD. J Nutr Biochem. (2011) 22:812–9. doi: 10.1016/j.jnutbio.2010.06.011 PMC3117068 21109419

[48] Magnone M Ameri P Salis A Andraghetti G Emionite L Murialdo G. Microgram amounts of abscisic acid in fruit extracts improve glucose tolerance and reduce insulinemia in rats and in humans. FASEB J Off Publ Fed Am Soc Exp Biol. (2015) 29:4783–93. doi: 10.1096/fj.15-277731 26243865

[49] Guri AJ Hontecillas R Si H Liu D Bassaganya-Riera J. Dietary abscisic acid ameliorates glucose tolerance and obesity-related inflammation in db/db mice fed high-fat diets. Clin Nutr Edinb Scotl. (2007) 26:107–16. doi: 10.1016/j.clnu.2006.07.008 17000034

[50] Atkinson FS Villar A Mulà A Zangara A Risco E Smidt CR. Abscisic acid standardized fig (Ficus carica) extracts ameliorate postprandial glycemic and insulinemic responses in healthy adults. Nutrients. (2019) 11:1757. doi: 10.3390/nu11081757 31370154 PMC6722713

[51] Magnone M Sturla L Guida L Spinelli S Begani G Bruzzone S. Abscisic acid: A conserved hormone in plants and humans and a promising aid to combat prediabetes and the metabolic syndrome. Nutrients. (2020) 12:1724. doi: 10.3390/nu12061724 32526875 PMC7352484

[52] Qi CC Ge JF Zhou JN. Preliminary evidence that abscisic acid improves spatial memory in rats. Physiol Behav. (2015) 139:231–9. doi: 10.1016/j.physbeh.2014.11.053 25449403

[53] Qi CC Zhang Z Fang H Liu J Zhou N Ge JF. Antidepressant effects of abscisic acid mediated by the downregulation of corticotrophin-releasing hormone gene expression in rats. Int J Neuropsychopharmacol. (2014) 18:pyu006. doi: 10.1093/ijnp/pyu006 25552429 PMC4360223

[54] Qi CC Shu YM Chen FH Ding YQ Zhou JN. Sensitivity during the forced swim test is a key factor in evaluating the antidepressant effects of abscisic acid in mice. Behav Brain Res. (2016) 300:106–13. doi: 10.1016/j.bbr.2015.12.009 26698394

[55] Sánchez-Sarasúa S Moustafa S García-Avilés Á López-Climent MF Gómez-Cadenas A Olucha-Bordonau FE. The effect of abscisic acid chronic treatment on neuroinflammatory markers and memory in a rat model of high-fat diet induced neuroinflammation. Nutr Metab. (2016) 13:73. doi: 10.1186/s12986-016-0137-3 PMC5081963 27795733

[56] Ribes-Navarro A Atef M Sánchez-Sarasúa S Beltrán-Bretones MT Olucha-Bordonau F Sánchez-Pérez AM. Abscisic acid supplementation rescues high fat diet-induced alterations in hippocampal inflammation and IRSs expression. Mol Neurobiol. (2019) 56:454–64. doi: 10.1007/s12035-018-1091-z 29721854

[57] Espinosa-Fernández V Mañas-Ojeda A Pacheco-Herrero M Castro-Salazar E Ros-Bernal F Sánchez-Pérez AM. Early intervention with ABA prevents neuroinflammation and memory impairment in a triple transgenic mice model of Alzheimer´s disease. Behav Brain Res. (2019) 374:112106. doi: 10.1016/j.bbr.2019.112106 31356828

[58] Guri AJ Hontecillas R Ferrer G Casagran O Wankhade U Noble AM. Loss of PPAR gamma in immune cells impairs the ability of abscisic acid to improve insulin sensitivity by suppressing monocyte chemoattractant protein-1 expression and macrophage infiltration into white adipose tissue. J Nutr Biochem. (2008) 19:216–28. doi: 10.1016/j.jnutbio.2007.02.010 17618105

[59] Kooshki R Anaeigoudari A Abbasnejad M Askari-Zahabi K Esmaeili-Mahani S. Abscisic acid interplays with PPARγ receptors and ameliorates diabetes-induced cognitive deficits in rats. Avicenna J Phytomedicine. (2021) 11:247–57. PMC8140211 34046321

[60] Sanchez-Perez AM. Abscisic acid, a promising therapeutic molecule to prevent Alzheimer’s and neurodegenerative diseases. Neural Regener Res. (2020) 15:1035–6. doi: 10.4103/1673-5374.270307 PMC7034262 31823879

[61] Bassaganya-Riera J Guri AJ Lu P Climent M Carbo A Sobral BW. Abscisic acid regulates inflammation via ligand-binding domain-independent activation of peroxisome proliferator-activated receptor gamma. J Biol Chem. (2011) 286:2504–16. doi: 10.1074/jbc.M110.160077 PMC3024745 21088297

[62] Li HH Hao RL Wu SS Guo PC Chen CJ Pan LP. Occurrence, function and potential medicinal applications of the phytohormone abscisic acid in animals and humans. Biochem Pharmacol. (2011) 82:701–12. doi: 10.1016/j.bcp.2011.06.042 21763293

[63] Jeon SH Kim N Ju YJ Gee MS Lee D Lee JK. Phytohormone abscisic acid improves memory impairment and reduces neuroinflammation in 5xFAD mice by upregulation of lanC-like protein 2. Int J Mol Sci. (2020) 21:8425. doi: 10.3390/ijms21228425 33182586 PMC7697599

[64] Afang Z Cui H Su W Liu C Shen L Yu X. Different doses of systemic LPS induce different degrees of polarization of microglia and astrocytes. Res Sq. (2021). doi: 10.21203/rs.3.rs-362185/v1

[65] Hussain R El-Etr M Gaci O Rakotomamonjy J Macklin WB Kumar N. Progesterone and Nestorone facilitate axon remyelination: a role for progesterone receptors. Endocrinology. (2011) 152:3820–31. doi: 10.1210/en.2011-1219 PMC6285137 21828184

[66] Meffre D Massaad C Grenier J. Lithium chloride stimulates PLP and MBP expression in oligodendrocytes via Wnt/β-catenin and Akt/CREB pathways. Neuroscience. (2015) 284:962–71. doi: 10.1016/j.neuroscience.2014.10.064 25451297

[67] Kaiser T Allen HM Kwon O Barak B Wang J He Z. MyelTracer: A semi-automated software for myelin g-ratio quantification. eNeuro. (2021) 8. ENEURO.0558-20.2021. doi: 10.1523/ENEURO.0558-20.2021 PMC8298095 34193510

[68] Mailleux J Timmermans S Nelissen K Vanmol J Vanmierlo T van Horssen J. Low-density lipoprotein receptor deficiency attenuates neuroinflammation through the induction of apolipoprotein E. Front Immunol. (2017) 8:1701. doi: 10.3389/fimmu.2017.01701 29276512 PMC5727422

[69] Maixner DW Christy D Kong L Viatchenko-Karpinski V Horner KA Hooks SB. Phytohormone abscisic acid ameliorates neuropathic pain via regulating LANCL2 protein abundance and glial activation at the spinal cord. Mol Pain. (2022) 18:17448069221107781. doi: 10.1177/17448069221107781 35647699 PMC9248043

[70] Kim SW Alci K Van Gaever F Driege Y Bicalho K Goeminne G. Engineering a highly sensitive biosensor for abscisic acid in mammalian cells. FEBS Lett. (2022) 596:2576–90. doi: 10.1002/1873-3468.14431 35727199

[71] Matsushima GK Morell P. The neurotoxicant, cuprizone, as a model to study demyelination and remyelination in the central nervous system. Brain Pathol Zurich Switz. (2001) 11:107–16. doi: 10.1111/j.1750-3639.2001.tb00385.x PMC8098267 11145196

[72] Gonsalvez DG Yoo S Fletcher JL Wood RJ Craig GA Murray SS. Imaging and quantification of myelin integrity after injury with spectral confocal reflectance microscopy. Front Mol Neurosci. (2019) 12:275. doi: 10.3389/fnmol.2019.00275 31803018 PMC6877500

[73] Guri AJ Hontecillas R Bassaganya-Riera J. Abscisic acid synergizes with rosiglitazone to improve glucose tolerance and down-modulate macrophage accumulation in adipose tissue: Possible action of the cAMP/PKA/PPAR γ axis. Clin Nutr. (2010) 29:646–53. doi: 10.1016/j.clnu.2010.02.003 PMC2888662 20207056

[74] Ebers GC Sadovnick AD Risch NJ. A genetic basis for familial aggregation in multiple sclerosis. Canadian Collaborative Study Group. Nature. (1995) 377:150–1. doi: 10.1038/377150a0 7675080

[75] Willer CJ Dyment DA Risch NJ Sadovnick AD Ebers GC Canadian Collaborative Study Group. Twin concordance and sibling recurrence rates in multiple sclerosis. Proc Natl Acad Sci U S A. (2003) 100:12877–82. doi: 10.1073/pnas.1932604100 PMC240712 14569025

[76] Farez MF Fiol MP Gaitán MI Quintana FJ Correale J. Sodium intake is associated with increased disease activity in multiple sclerosis. J Neurol Neurosurg Psychiatry. (2015) 86:26–31. doi: 10.1136/jnnp-2014-307928 25168393 PMC12930402

[77] Timmermans S Bogie JFJ Vanmierlo T Lütjohann D Stinissen P Hellings N. High fat diet exacerbates neuroinflammation in an animal model of multiple sclerosis by activation of the Renin Angiotensin system. J Neuroimmune Pharmacol Off J Soc NeuroImmune Pharmacol. (2014) 9:209–17. doi: 10.1007/s11481-013-9502-4 24068577

[78] Cheng-Chung Wei J Huang HC Chen WJ Huang CN Peng CH Lin CL. Epigallocatechin gallate attenuates amyloid β-induced inflammation and neurotoxicity in EOC 13.31 microglia. Eur J Pharmacol. (2016) 770:16–24. doi: 10.1016/j.ejphar.2015.11.048 26643169

[79] Endale M Park SC Kim S Kim SH Yang Y Cho JY. Quercetin disrupts tyrosine-phosphorylated phosphatidylinositol 3-kinase and myeloid differentiation factor-88 association, and inhibits MAPK/AP-1 and IKK/NF-κB-induced inflammatory mediators production in RAW 264.7 cells. Immunobiology. (2013) 218:1452–67. doi: 10.1016/j.imbio.2013.04.019 23735482

[80] Rothwell JA Perez-Jimenez J Neveu V Medina-Remón A M’hiri N García-Lobato P. Phenol-Explorer 3.0: a major update of the Phenol-Explorer database to incorporate data on the effects of food processing on polyphenol content. Database J Biol Database Curation. (2013) 2013:bat070. doi: 10.1093/database/bat070 PMC3792339 24103452

[81] Garcia Corrales AV Verberk SGS Haidar M Grajchen E Dehairs J Vanherle S. Fatty acid elongation by ELOVL6 hampers remyelination by promoting inflammatory foam cell formation during demyelination. Proc Natl Acad Sci U S A. (2023) 120:e2301030120. doi: 10.1073/pnas.2301030120 37669365 PMC10500284

[82] Savage JC Carrier M Tremblay MÈ. Morphology of microglia across contexts of health and disease. Methods Mol Biol Clifton NJ. (2019) 2034:13–26. doi: 10.1007/978-1-4939-9658-2_2 31392674

[83] Plastini MJ Desu HL Brambilla R. Dynamic responses of microglia in animal models of multiple sclerosis. Front Cell Neurosci. (2020) 14:269. doi: 10.3389/fncel.2020.00269 32973458 PMC7468479

[84] Heppner FL Greter M Marino D Falsig J Raivich G Hövelmeyer N. Experimental autoimmune encephalomyelitis repressed by microglial paralysis. Nat Med. (2005) 11:146–52. doi: 10.1038/nm1177 15665833

[85] Constantinescu CS Farooqi N O’Brien K Gran B. Experimental autoimmune encephalomyelitis (EAE) as a model for multiple sclerosis (MS). Br J Pharmacol. (2011) 164:1079–106. doi: 10.1111/j.1476-5381.2011.01302.x PMC3229753 21371012

[86] Sokołowska P Bleibel L Owczarek J Wiktorowska-Owczarek A. PPARγ, NF-κB and the UPR pathway as new molecular targets in the anti-inflammatory actions of NSAIDs: Novel applications in cancers and central nervous system diseases? Adv Clin Exp Med Off Organ Wroclaw Med Univ. (2024) 33(9):1007–22. doi: 10.17219/acem/174243 38180328

[87] Schoonjans K Staels B Auwerx J. The peroxisome proliferator activated receptors (PPARS) and their effects on lipid metabolism and adipocyte differentiation. Biochim Biophys Acta. (1996) 1302:93–109. doi: 10.1016/0005-2760(96)00066-5 8695669

[88] Hontecillas R Bassaganya-Riera J. Expression of PPAR γ in intestinal epithelial cells is dispensable for the prevention of colitis by dietary abscisic acid. E-SPEN J. (2012) 7:e189–95. doi: 10.1016/j.clnme.2012.07.002 PMC3691880 23814701

[89] Moreno S Farioli-Vecchioli S Cerù MP. Immunolocalization of peroxisome proliferator-activated receptors and retinoid X receptors in the adult rat CNS. Neuroscience. (2004) 123:131–45. doi: 10.1016/j.neuroscience.2003.08.064 14667448

[90] Cheng H Shang Y Jiang L lu ST Wang L. The peroxisome proliferators activated receptor-gamma agonists as therapeutics for the treatment of Alzheimer’s disease and mild-to-moderate Alzheimer’s disease: a meta-analysis. Int J Neurosci. (2016) 126:299–307. doi: 10.3109/00207454.2015.1015722 26001206

[91] Masciopinto F Di Pietro N Corona C Bomba M Pipino C Curcio M. Effects of long-term treatment with pioglitazone on cognition and glucose metabolism of PS1-KI, 3xTg-AD, and wild-type mice. Cell Death Dis. (2012) 3:e448. doi: 10.1038/cddis.2012.189 23254291 PMC3542623

[92] Loix M Wouters E Vanherle S Dehairs J McManaman JL Kemps H. Perilipin-2 limits remyelination by preventing lipid droplet degradation. Cell Mol Life Sci CMLS. (2022) 79:515. doi: 10.1007/s00018-022-04547-0 36100764 PMC11803036

[93] Moore KJ Rosen ED Fitzgerald ML Randow F Andersson LP Altshuler D. The role of PPAR-gamma in macrophage differentiation and cholesterol uptake. Nat Med. (2001) 7:41–7. doi: 10.1038/83328 11135614

