# Corrigendum: The phytohormone abscisic acid enhances remyelination in mouse models of multiple sclerosis

**DOI:** 10.3389/fimmu.2025.1562292

**Published:** 2025-02-07

**Authors:** Femke Van Gaever, Fleur Mingneau, Sam Vanherle, Yasmine Driege, Mira Haegman, Elien Van Wonterghem, Junhua Xie, Roosmarijn E. Vandenbroucke, Jerome J. A. Hendriks, Rudi Beyaert, Jens Staal

**Affiliations:** ^1^ VIB-UGent Center for Inflammation Research, VIB, Ghent, Belgium; ^2^ Department of Biomedical Molecular Biology, Ghent University, Ghent, Belgium; ^3^ Department of Immunology and Infection, Biomedical Research Institute, Hasselt University, Diepenbeek, Belgium; ^4^ University MS Center Hasselt, Pelt, Belgium; ^5^ Department of Biochemistry and Microbiology, Ghent University, Ghent, Belgium

**Keywords:** abscisic acid, remyelination, macrophages, microglia, multiple sclerosis, myelin, neuroinflammation

In the published article, there was an error in the author list, and author Rudi Beyaert was erroneously excluded from being listed as shared last author. The corrected author list appears below.

“Femke Van Gaever, Fleur Mingneau, Sam Vanherle, Yasmine Driege, Mira Haegman, Elien Van Wonterghem, Junhua Xie, Roosmarijn E. Vandenbroucke, Jerome J. A. Hendriks, Rudi Beyaert^*†^, Jens Staal^*†^

*Correspondence: Rudi Beyaert rudi.beyaert@irc.vib-ugent.be Jens Staal jens.staal@irc.vib-ugent.be

^†^These authors share last authorship”

The authors apologize for this error and state that this does not change the scientific conclusions of the article in any way. The original article has been updated.

