# Supplementary figures and images for: The phytohormone abscisic acid enhances remyelination in mouse models of multiple sclerosis
Source: Front Immunol. 2024 Dec 17;15:1500697. doi: 10.3389/fimmu.2024.1500697 (PMC11685095; doi:10.3389/fimmu.2024.1500697)

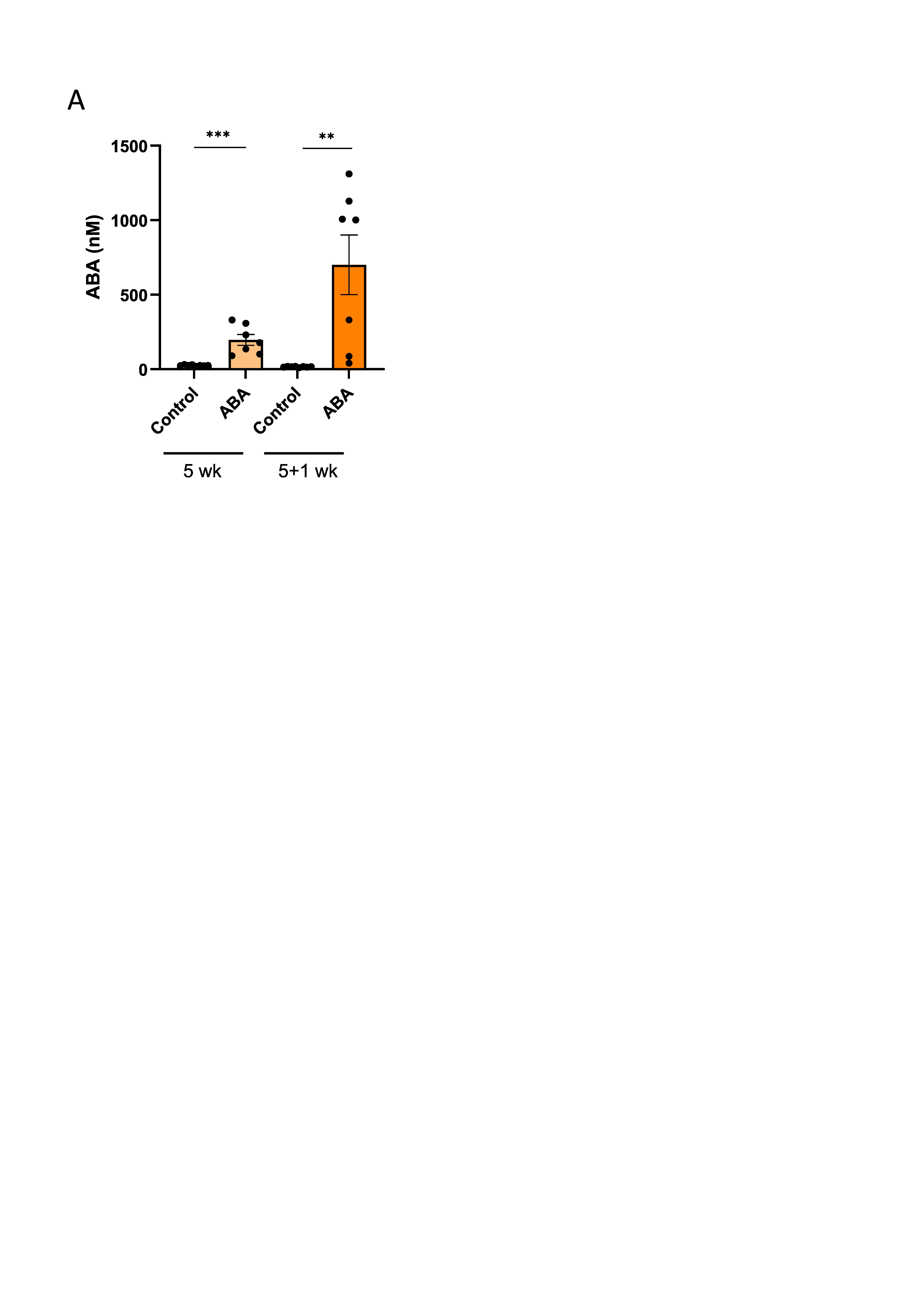

Supplement: Supplementary Figure 1 — ABA is increased in serum from ABA-fed mice. (A) ABA measurement in serum from mice given a cuprizone supplemented control or ABA diet (400 mg/kg). Serum was taken at sacrifice, after 5 weeks for the demyelination group and after 5 + 1 weeks for the remyelination group. ABA is measured by PYL1H87P-based biosensor. Each dot represents one mouse (n = 7-8). Data are represented as mean ± SEM and statistically analyzed using a Student’s t-test. **p<0.01, ***p<0.001. [file Image1.jpeg]

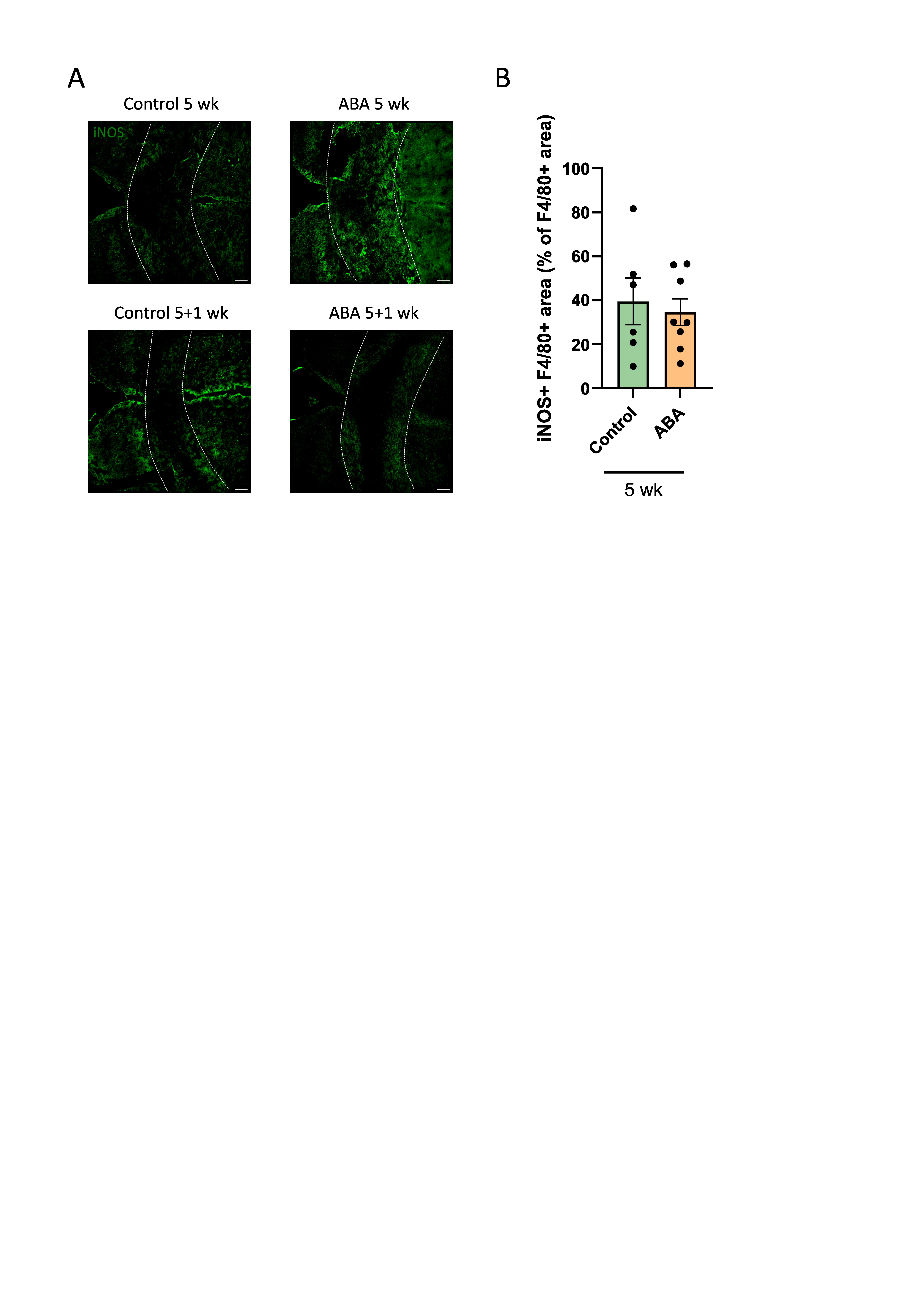

Supplement: Supplementary Figure 2 — Measurement of iNOS staining. (A) Representative images of immunofluorescent iNos staining of the corpus callosum from mice fed with control diet or ABA-supplemented diet. The outer border of the corpus callosum is demarcated by the dotted line. Scale bars, 200 μm. (B) Quantification of the percentage iNos+ F4/80+ area out of total F4/80+ area in the corpus callosum from cuprizone animals fed with control diet or ABA diet (n = 6-8 animals; 2 images per animal). Each dot represents one mouse. Data are represented as mean ± SEM and statistically analyzed using a Student’s t-test. [file Image2.jpeg]

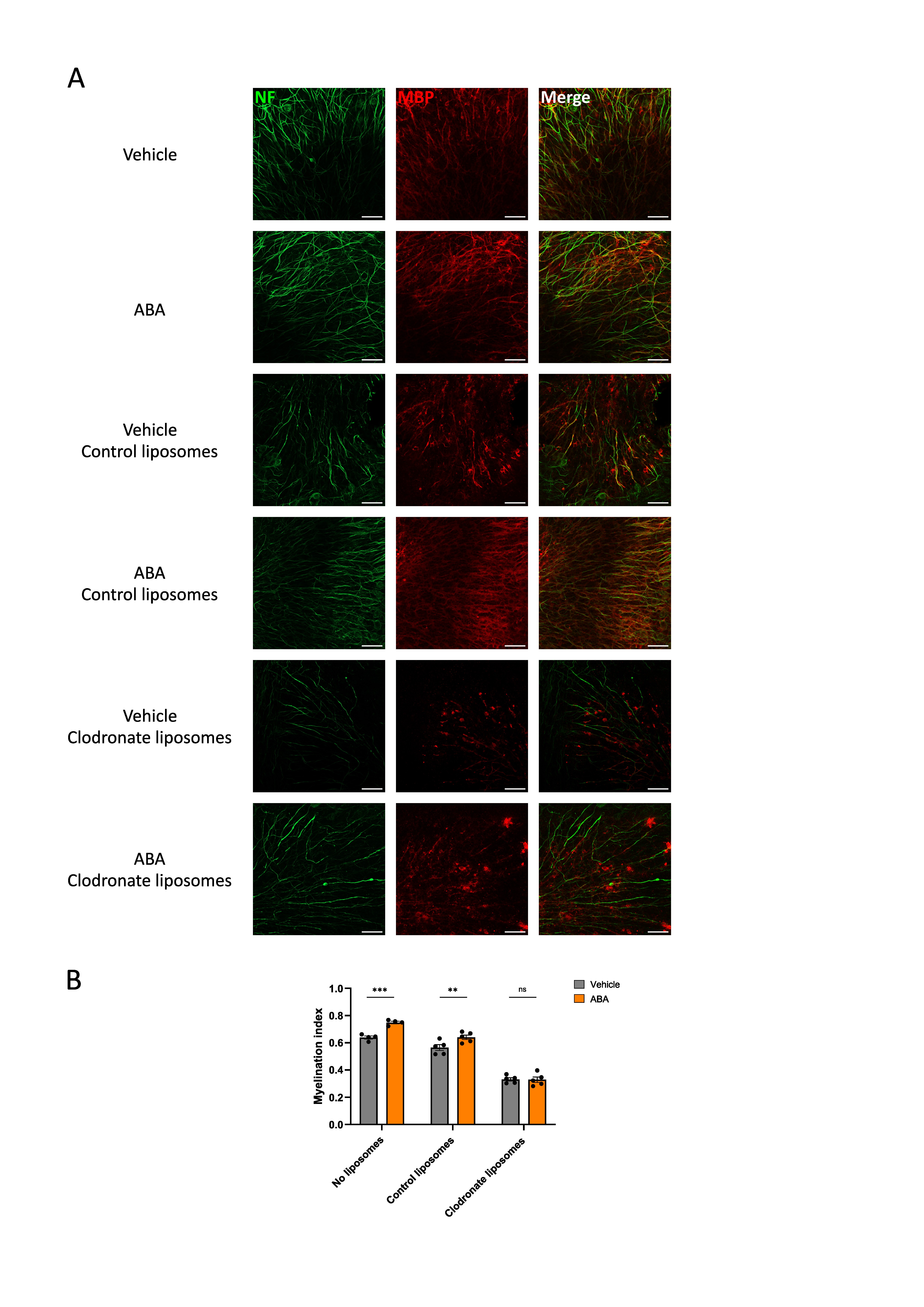

Supplement: Supplementary Figure 3 — Measurement of remyelination efficiency in microglia-depleted brain slice cultures. (A) Representative images of immunofluorescent MBP/NF stains of cerebellar brain slices treated with vehicle or ABA, without lipsomes, with empty liposomes (control liposomes) or clodronate liposomes. Scale bars, 50 μm. (B) Relative number of MBP+ NF+ axons out of total NF+ axons in cerebellar brain slices stimulated with control liposomes or clodronate liposomes (0.5 mg/mL) and treated with vehicle or ABA (n = 3 slices). Each dot represents one slice. Data are represented as mean ± SEM and statistically analyzed using a Student’s t-test. *p<0.05, **p<0.01. [file Image3.jpeg]

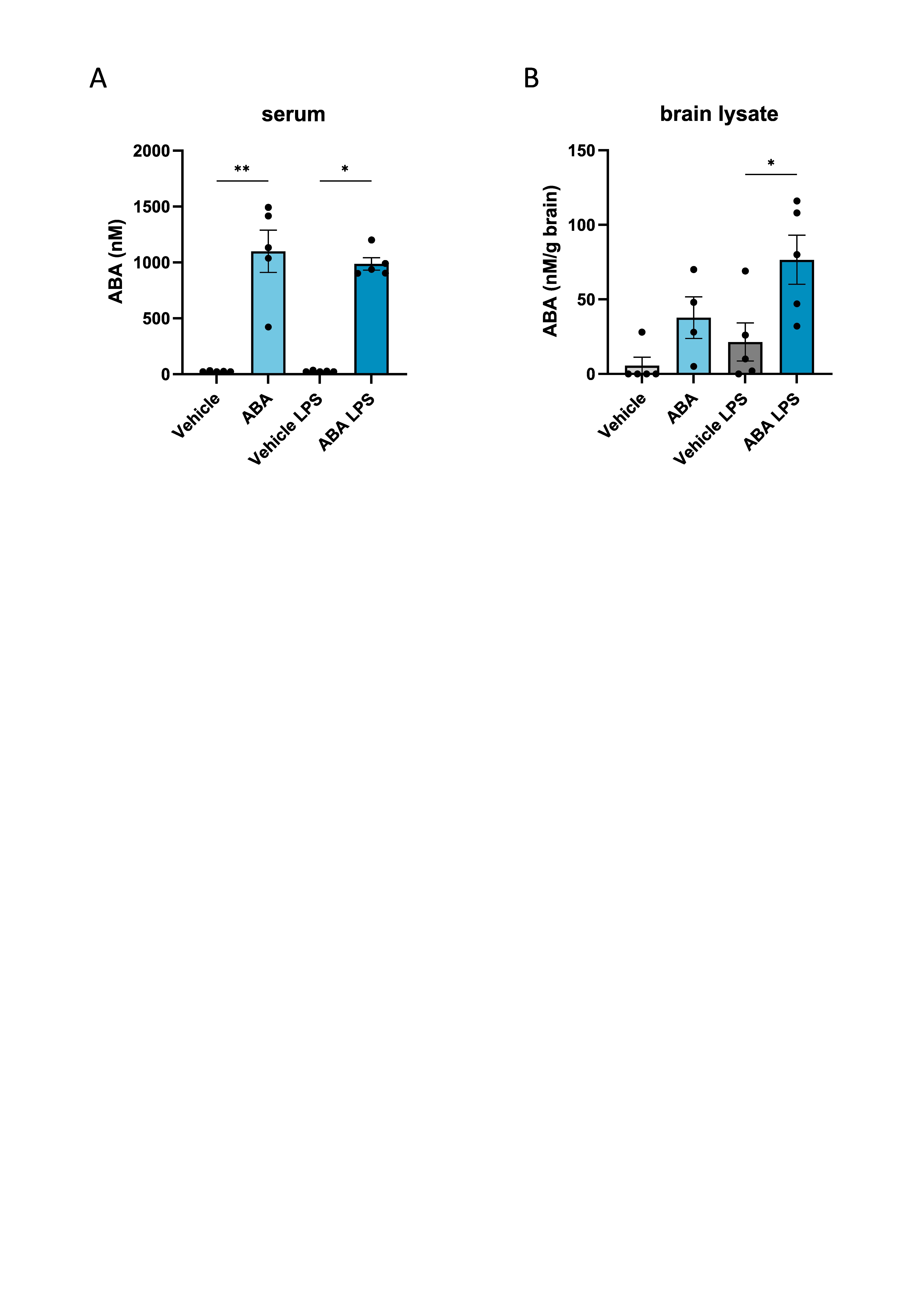

Supplement: Supplementary Figure 4 — Measurement of ABA in serum and brain lysates from mice in the LPS model. (A-B) ABA measurement in serum (A) and brain lysate (B) from mice fed a control or ABA-supplemented diet and subjected to LPS injection. ABA is measured by PYL1H87P-based biosensor. Each dot represents one mouse. Data are represented as mean ± SEM and statistically analyzed using a Student’s t-test. *p<0.05, **p<0.01. [file Image4.jpeg]

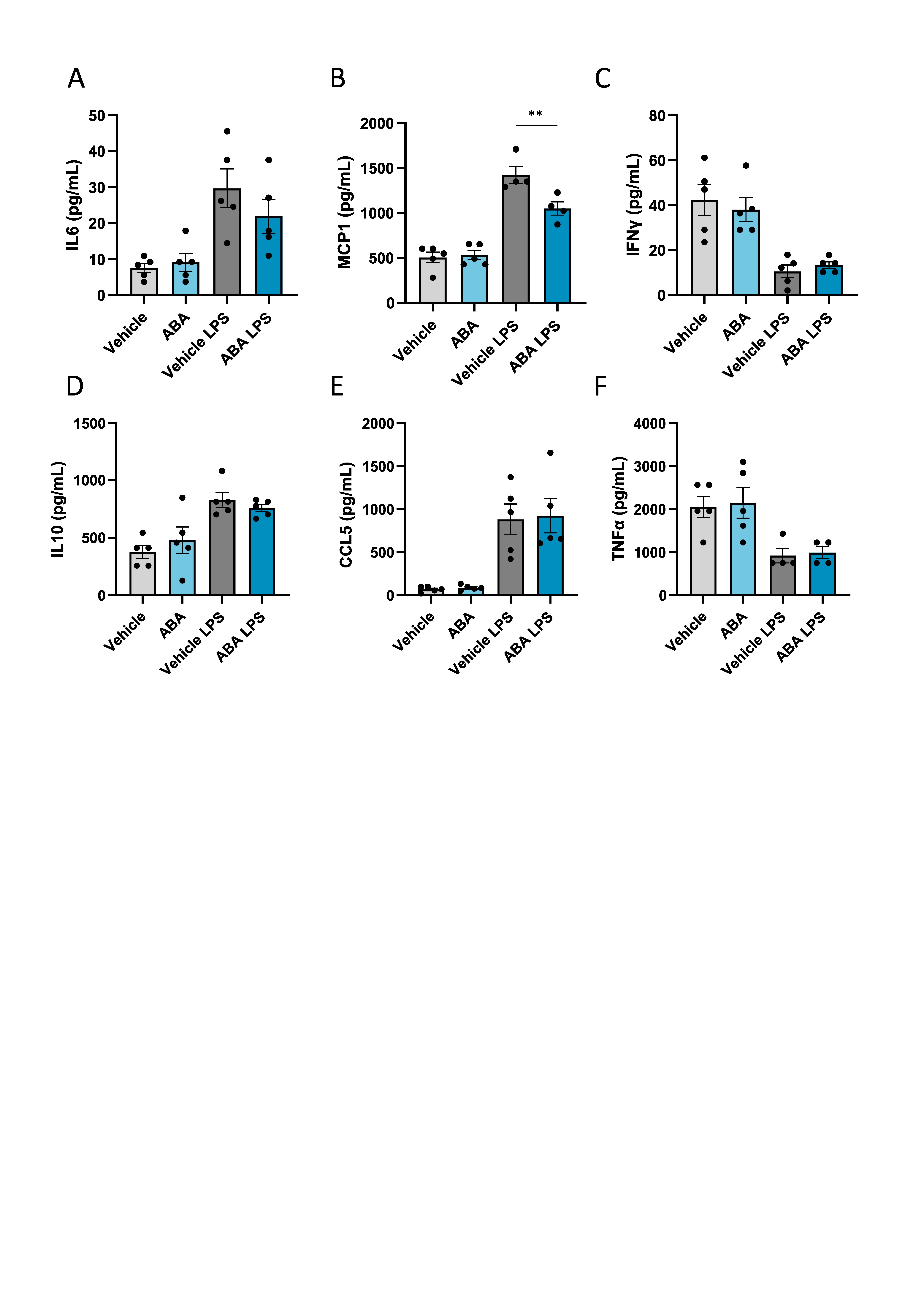

Supplement: Supplementary Figure 5 — Cytokine and chemokine measurements in serum from mice in the LPS model. (A-F) Protein levels of IL6 (A), MCP1 (B), IFNγ (C), IL10 (D), CCL5 (E), and TNFα (F) in plasma as measured by Bioplex (n = 5). Each dot represents one mouse (n = 5). Data are represented as mean ± SEM and statistically analyzed using a one-way ANOVA with correction for multiple testing. *p < 0.05, **p < 0.01, ***p<0.001, ****p<0.0001. [file Image5.jpeg]

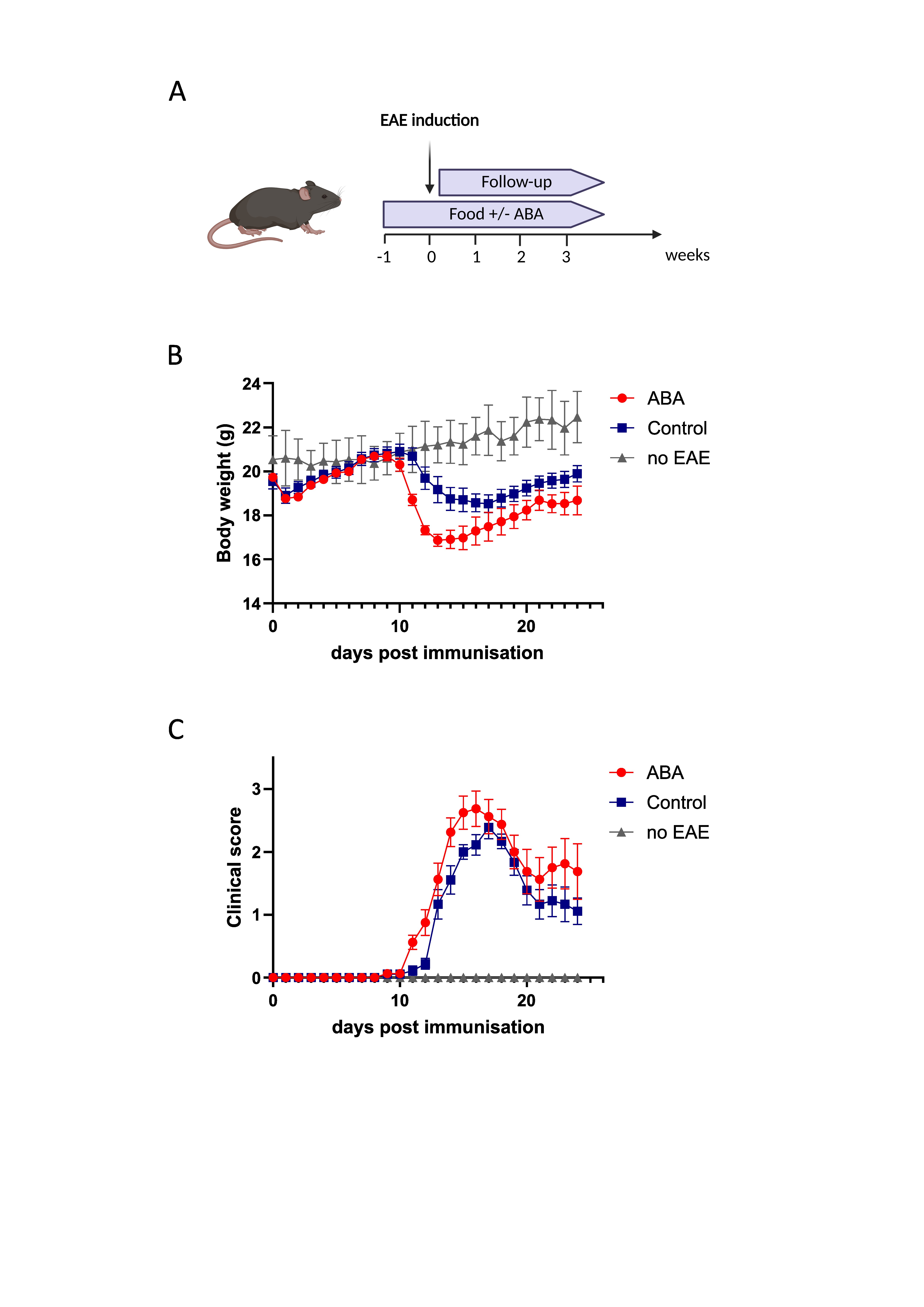

Supplement: Supplementary Figure 6 — ABA has no effect on disease progression in an experimental autoimmune encephalomyelitis (EAE) model. (A) Schematic representation of the experimental design: mice were treated with control or ABA-supplemented diet starting 1 week before EAE induction and continued until the end of the experiment. From the day of immunization, daily measurements of body weight and clinical disease symptoms was performed. Created with biorender.com. (B, C) Body weight (g) measurements (B) and clinical disease scores (C) of ABA treated EAE mice versus control EAE mice (n = 8). [file Image6.jpeg]

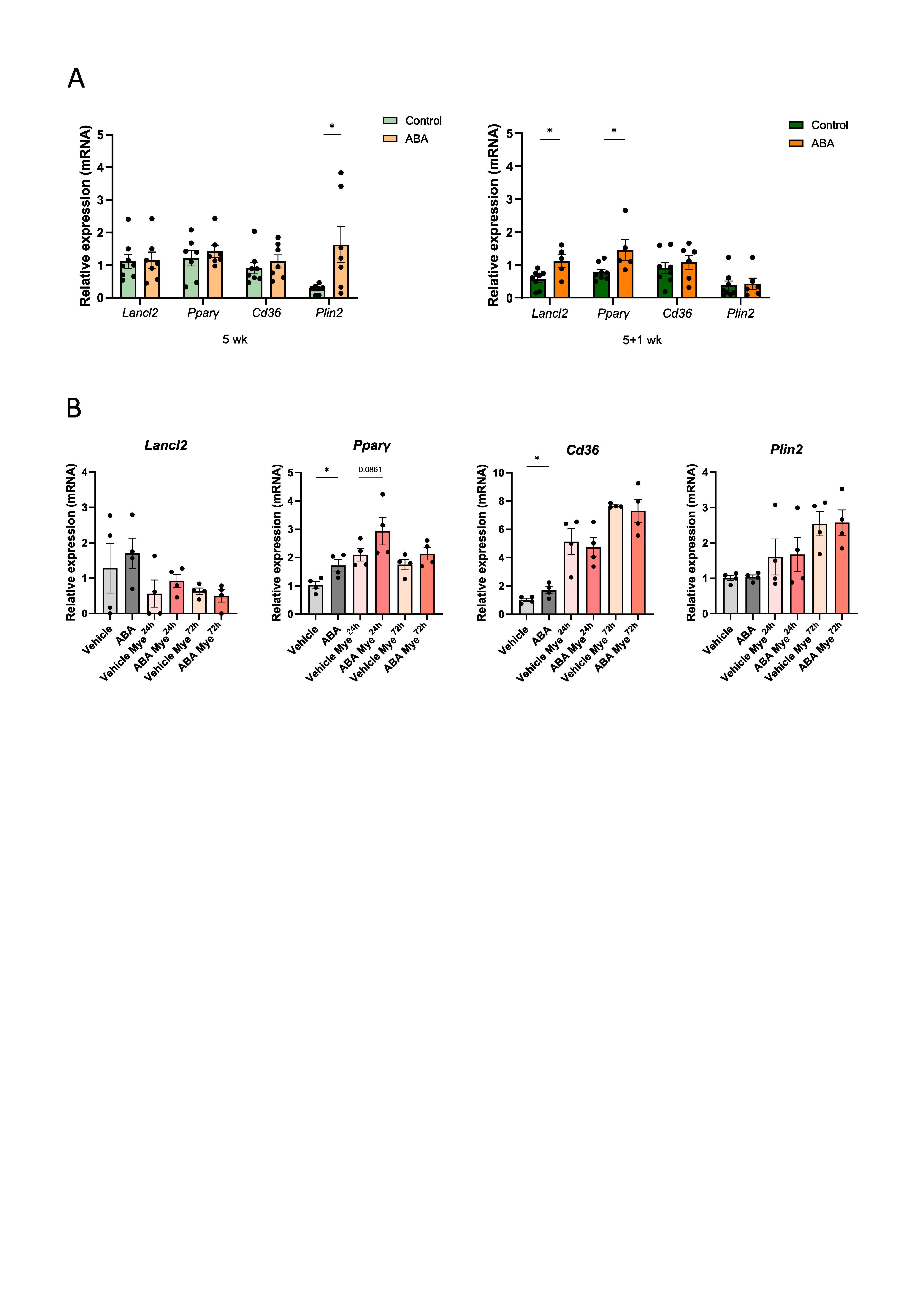

Supplement: Supplementary Figure 7 — ABA affects mRNA expression of Lancl2, Pparγ, Cd36 and Plin2. (A) mRNA expression of Lancl2, Pparγ, Cd36 and Plin2 in the corpus callosum from control-fed and ABA-fed mice after 5 weeks and 5 + 1 weeks of cuprizone treatment (n = 7–8 animals). Each dot represents one mouse. One outlier was removed for Plin2 after ROUT method of detecting outliers with Q = 0.1% (Plin2: n = 7). (B) mRNA expression of Lancl2, Pparγ, Cd36 and Plin2 in BMDMs (n = 5) exposed to vehicle or ABA and treated with myelin for 0, 24 or 72 hours. Each dot represents one well. Data are represented as mean ± SEM and statistically analyzed using a Student’s t-test. *p<0.05. [file Image7.jpeg]
