# Supplementary material for: The phytohormone abscisic acid enhances remyelination in mouse models of multiple sclerosis
Source: Front Immunol. 2024 Dec 17;15:1500697. doi: 10.3389/fimmu.2024.1500697 (PMC11685095; doi:10.3389/fimmu.2024.1500697)
Supplement: Supplementary file 8 [file Table1.docx]

**Table S1. qPCR primers**

| **Gene** | **Primer** | **Sequence** |
| --- | --- | --- |
| *Rlp13* | *Rlp13* Forward | CCTGCTGCTCTCAAGGTT |
|  | *Rlp13* Reverse | TGGTTGTCACTGCCTGGTACTT |
| *Tbp* | *Tbp* Forward | TCTACCGTGAATCTTGGCTGTAAA |
|  | *Tbp* Reverse | TTCTCATGATGACTGCAGCAAA |
| *Gapdh* | *Gapdh* Forward | TGGCAACAATCTCCACTTTGC |
|  | *Gapdh* Reverse | ACAAAATGGTGAAGGTCGGTG |
| *Sdha* | *Sdha* Forward | GGAACACTCCAAAAACAGACCT |
|  | *Sdha* Reverse | CCACCACTGGGTATTGAGTAGAA |
| *Ywhaz* | *Ywhaz* Forward | GAAAAGTTCTTGATCCCCAATGC |
|  | *Ywhaz* Reverse | TGTGACTGGTCCACAATTCCTT |
| *Mbp* | *Mbp* Forward | TCACAGAAGAGACCCTCACAGC |
|  | *Mbp* Reverse | GAGTCAAGCATGCCCGTGTC |
| *Plp* | *Plp* Forward | TTGTTTGGGAAAATGGCTAGG |
|  | *Plp* Reverse | GCAGATCGACAGAAGCTTGGA |
| *Ccl4* | *Ccl4* Forward | TTCCTGCTGTTTCTCTTACACCT |
|  | *Ccl4* Reverse | CTGTCTGCCTCTTTTGGTCAG |
| *Ccl5* | *Ccl5* Forward | CGTCAAGGAGTATTTCTACAC |
|  | *Ccl5* Reverse | GGTCAGAATCAAGAAACCCT |
| *Cntf* | *Cntf* Forward | TCTGTAGCCGCTCTATCTGG |
|  | *Cntf* Reverse | GGTACACCATCCACTGAGTCAA |
| *Igf1* | *Igf1* Forward | TACTTCAACAAGCCCACAGGC |
|  | *Igf1* Reverse | ATAGAGCGGGCTGCTTTTGT |
| *Ngf* | *Ngf* Forward | GGAGCGCATCGAGTTTTGG |
|  | *Ngf* Reverse | TCCTTGGCAAAACCTTTATTGGG |
| *Tgfβ* | *Tgfβ* Forward | GGGCTACCATGCCAACTTCTG |
|  | *Tgfβ* Reverse | GAGGGCAAGGACCTTGCTGTA |
| *Tnfα* | *Tnfα* Forward | CCAGACCCTCACACTCAG |
|  | *Tnfα* Reverse | CACTTGGTGGTTTGCTACGAC |
| *Lancl2* | *Lancl2* Forward | CCCAACTTCATAAAGCGTATCCA |
|  | *Lancl2* Reverse | GCAGGTACAAAAGTGCTATGCC |
| *Pparγ* | *Pparγ* Forward | CAGGCTTGCTGAACGTGAAG |
|  | *Pparγ* Reverse | GGAGCACCTTGGCGAACA |
| *Cd36* | *Cd36* Forward | GGACATTGAGATTCTTTTCCTCTG |
|  | *Cd36* Reverse | GCAAAGGCATTGGCTGGAAGAAC |
| *Plin2* | *Plin2* Forward | GACAGGATGGAGGAAAGACTGC |
|  | *Plin2* Reverse | GGTAGTCGTCACCACATCCTTC |
