# Supplementary material for: The phytohormone abscisic acid enhances remyelination in mouse models of multiple sclerosis
Source: Front Immunol. 2024 Dec 17;15:1500697. doi: 10.3389/fimmu.2024.1500697 (PMC11685095; doi:10.3389/fimmu.2024.1500697)
Supplement: Supplementary file 9 [file DataSheet1.pdf]

Supplementary Material

1 Supplementary Figures

Suppl. Figure 1: Example assay clinical and technical validation. For clinical validation, 231 samples were measured with four commercial ELISAs for EA IgG (**A-B**), VCA IgG (**D-E**), VCA IgM (**G-H**) and EBNA1 IgG (**J-K**) and compared with the multiplex assay, with Pearson's correlation used to determine agreement. Data is presented as both a regression comparing Multiplex Immunoassay MFI values to ELISA RU (A, D, G, J), and as scatterplots of multiplex immunoassay data based upon sample classification determined by the ELISA (B, E, H, K). For technical validation, 4 samples were measured in duplicate (mean is shown) across 5 plates (**C, F, I, L**) to assess inter-assay variation, with all CVs <20% indicating stable assay performance.

Suppl. Figure 2: Demographic data including **(A)** age, **(B)** disease duration, **(C)** EDSS (Expanded Disability Status Scale), and **(D)** number of previous treatments is shown for relapsing remitting multiple sclerosis patients during relapse (RRMS RE), untreated and without relapse (RRMS UT), untreated secondary progressive multiple sclerosis (SPMS UT) and untreated primary progressive multiple sclerosis (PPMS) and when applicable EBV neg. controls, EBV pos. controls. Kruskal-Wallis test with Dunn's correction was applied for statistical testing (* $p < 0.05$, ** $p < 0.01$, *** $p < 0.001$, **** $p < 0.0001$).

Suppl. Figure 3: Demographic data including **(A)** age, **(B)** disease duration, **(C)** EDSS (Expanded Disability Status Scale), **(D)** number of previous treatments, and **(E)** treatment duration is shown for relapsing remitting multiple sclerosis patients (RRMS) without treatment (UT), and during treatment with glatiramer acetate (GLAT), teriflunomide (TER), dimethyl fumarate (DMF), cladribine (CLAD), ozanimod (OZA), natalizumab (NAT), and ocrelizumab (OCR). Kruskal-Wallis test with Dunn's correction was applied for statistical testing (* $p < 0.05$, ** $p < 0.01$, *** $p < 0.001$, **** $p < 0.0001$).

Suppl. Figure 4: Treatment effects on antibody titers against lifecycle proteins/ peptides. Mean fluorescence intensities (MFI) of the serum antibody response against **(A)** gp350/220 docking protein, **(B)** gH/gp42 and **(C)** gh/gL/gp42 fusion proteins, **(D)** EA P85, **(E)** EA P138, **(F)** EA P54 early antigens, **(G)** VCA P18, **(H)** VCA P23, and **(I)** VCA gp125 viral capsid antigens **(J)** EBNA1 late antigen, and **(K)** BZLF1 immediate early antigen, are shown for relapsing remitting multiple sclerosis patients (RRMS) without treatment (UT), and during treatment with glatiramer acetate (GLAT), teriflunomide (TER), dimethyl fumarate (DMF), cladribine (CLAD), ozanimod (OZA), natalizumab (NAT), and ocrelizumab (OCR). Kruskal-Wallis test with Dunn's correction was applied for statistical testing .

Suppl. Figure 5: Correlation matrix between EBV lifecycle proteins and demographic data. The Spearman correlation test was employed for the purpose of statistical analysis. DMTs = disease-modifying treatments; EDSS = Expanded Disability Status Scale.

Suppl. Figure 6: Treatment effects on EBNA1 peptides and cross-reactive peptides. Mean fluorescence intensities (MFI) of the serum antibody response against **(A)** EBNA1 full antigen, **(B)** EBNA1 p72 (Serion), **(C)** EBNA1 p72 (Aviva), **(D)** EBNA1 AA386-405, **(E)** EBNA1 AA393-412, **(F)** EBNA1 AA425-444, **(G)** GlialCAM AA370-389, **(H)** CRYAB AA2-21, and **(I)** ANO2 AA134-153 are shown for relapsing remitting multiple sclerosis patients (RRMS) without treatment (UT), and

during treatment with glatiramer acetate (GLAT), teriflunomide (TER), dimethyl fumarate (DMF), cladribine (CLAD), ozanimod (OZA), natalizumab (NAT), and ocrelizumab (OCR). Kruskal-Wallis test with Dunn's correction was applied for statistical testing.
